# Parasitic diarrheal disease: drug development and targets

**DOI:** 10.3389/fmicb.2015.01183

**Published:** 2015-10-27

**Authors:** Amir Azam, Mudasir N. Peerzada, Kamal Ahmad

**Affiliations:** ^1^Medicinal Chemistry Laboratory, Department of Chemistry, Jamia Millia IslamiaNew Delhi, India; ^2^Centre for Interdisciplinary Research in Basic Sciences, Jamia Millia IslamiaNew Delhi, India

**Keywords:** diarrhea, causative parasitic agents, chemotherapy, drug targets, therapeutic developments

## Abstract

Diarrhea is the manifestation of gastrointestinal infection and is one of the major causes of mortality and morbidity specifically among the children of less than 5 years age worldwide. Moreover, in recent years there has been a rise in the number of reports of intestinal infections continuously in the industrialized world. These are largely related to waterborne and food borne outbreaks. These occur by the pathogenesis of both prokaryotic and eukaryotic organisms like bacteria and parasites. The parasitic intestinal infection has remained mostly unexplored and under assessed in terms of therapeutic development. The lack of new drugs and the risk of resistance have led us to carry out this review on drug development for parasitic diarrheal diseases. The major focus has been depicted on commercially available drugs, currently synthesized active heterocyclic compounds and unique drug targets, that are vital for the existence and growth of the parasites and can be further exploited for the search of therapeutically active anti-parasitic agents.

## Introduction

Diarrhea is a symptom of an infection in the intestinal tract, which can be caused by variety of bacterial, viral, and parasitic organisms. Infection spreads through contaminated food or drinking water or from person to person as a result of poor hygiene. Diarrheal diseases is the second leading cause of death in children under 5 years and is responsible for killing around 760,000 children every year (World Health Organization, 2013). The common symptoms include the frequent bowel movements, loose watery stool, incontinence, lower abdominal pain, or cramping and in severe cases it causes blood or flecks of mucus in the stool. The dreadful infection leads to the loss of appetite and patient is more likely to suffer from loss of weight. There are a number of non-infectious medical conditions that may cause diarrhea too. The inability in digesting dairy products which includes lactose intolerance, coeliac disease which is an intolerance of gluten in wheat and some other grains and pancreatic problems (cystic fibrosis) which interfere with production of important digestive substances (Haque et al., 2003). Several agents that cause diarrhea include viruses (rotavirus or Norwalk virus, enterovirus, or a hepatitis virus), bacteria (*Shigella* species, *Escherichia coli*, and *Campylobacter jejuni*) and parasites (*Giardia lamblia, Entamoeba histolytica*, and *Cryptosporidium* species; Shah et al., 2009). Diarrhea caused by parasites is unlike that of either bacterial or viral infections. For instance, *Giardia* has a slow onset of diarrhea and can be present for months, while most bacterial and viral infections are limited to 1–2 weeks (Petri, 2003). In addition, parasites are eukaryotic, which makes them larger and more complex than either viruses or bacteria and also more difficult to eradicate due to their similarity to the host. Though the infection is caused by a number of parasites which mainly include *G. lamblia, E. histolytica, Cryptosporidium parvum, Cyclospora cayetanensis, Isospora belli, Blastocystis hominis* but the enzyme immunoassays are only available for the testing of three parasites *G. lamblia, E. histolytica*, and *C. parvum* (Slack, 2012).

Many of the new drug targets are being discovered with much more ease as the genomic sequences of many parasitic organisms are becoming available. This advancement has enabled researchers not only to identify new biochemical pathways and gene families that can be short-listed as potential drug targets but also has significantly increased their basic understanding of parasites that cause some of the most severe diseases. Moreover, biochemical analysis and genome sequencing both have helped in identifying potential targets, enzymes, transporters, and metabolites that are distinct in parasites and their mammalian host (Loftus et al., 2005). As a consequence one can design even better and safer effective drugs that can be used in future to combat the disease caused by the organism. The heterocyclic compounds with different modifications have been synthesized and their biological evaluation have identified them to fight the diarrhea causing parasites. In the following discussion some of the most important parasites causing diarrhea, their drugs, important traced targets and the active heterocyclic compounds synthesized against them have been summarized.

## Microsporidia

*Enterocytozoon bieneusi, Encephalitozoon cuniculi*, and *Encephalitozoon intestinalis* are the reported species of microsporidia which are the causative agents of chronic diarrhea predominantly in immunocompromised individuals such as AIDS patients (Blanshard et al., 1992; Chokephaibulkit et al., 2001; Goodgame, 2003). The diarrheal disease caused by microsporodia spp. is treated by drugs fumagilin (**1)** and albendazole (**2**) (Goodgame, 2003; Agholi et al., 2013). The polyamine analogs, fumagillin-related compounds and analogs, nikkomycins **(3**), fluoroquinolones (**4–9**) and benzimidazole-related compounds are the active sources of new compounds against microsporidiosis. Fumagillin, a natural product (**1**) is an antibiotic and anti-angiogenic compound produced by *Aspergillus fumigatus* and has been found potent against Encephalitozoon spp. and *E. bieneusi in vitro* (Bacchi and Weiss, 2004). The antimicrosporidial activity of various fluoroquinolones derivatives such as norfloxacin (**4**) and ofloxacin (**5**) is more than gatifloxacin (**6**), lomefloxacin (**7**), moxifloxacin (**8**), and nalidixic acid (**9**) and can be the effective option of chemotherapy (Didier et al., 2005). It was observed that TNP-470 (**10**), ovalicin (**11**) and its derivatives inhibited *E. intestinalis* replication by more than 70% *in vitro* which indicates that these compounds can be used as medication for this infection (Didier et al., 2006). It has been also reported that the use of chlorine and ozone as disinfectants kills the *E. intestinalis* in water (John et al., 2005).

The albendazole, a benzimidazole derivative inhibits the microtubule assembly in *E*. *intestinalis* but not in *E. bieneusi* infections because benzimidazoles bind to the colchicine-binding site of β-tubulin monomer prior to dimerization with α-tubulin which blocks subsequent microtubule formation (Macdonald et al., 2004). The β-tubulin subunit is the primary target of benzimidazole, where its predictive sensitive residues are Cys 165, Phe 167, Glu 198, Phe 200, Arg 242, and Val 268 (Macdonald et al., 2004; Tremoulet et al., 2004). It has been also reported that microtubule assembly gets inhibited due to the presence of hydrogen atom at the 1-position and a methoxycarbonylamino group at the 2-position of the benzimidazole ring (Friedman and Platzer, 1980). Fumagillin along with its analogs act on the methionine aminopeptidase type 2(MetAP2) by non-competitive inhibition, i.e., by irreversibly blocking the active site in *E. cuniculi* and it seems to be the potent inhibitor (Molina et al., 2002). Analysis of EcMetAP2 demonstrated conservation of the key residues associated with the active site of this class of enzymes including Asp251, Asp262, His331, Glu364, and Glu459 (involved in coordination of metal binding); His231 (to which fumagillin covalently binds); and Phe219, Leu328, Ile338, His339, Asp378, Tyr444, Leu447 (involved in binding substrates into the active site; Upadhya et al., 2006). The aspartic proteases are a family of protease enzymes that use two highly conserved aspartic acid residues in the active site for catalytic cleavage of their peptide substrates (Pozio and Morales, 2005). Ritonavir (**12**) and indinavir (**13**) drugs are inhibitors of aspartyl protease, used in the highly-active antiretroviral therapy cocktail, inhibited the growth of *E. intestinalis* in tissue culture due to intermolecular hydrogen bonding between -NH group of these drugs with aspartic acid residue (Menotti et al., 2005; Pozio and Morales, 2005). The polyamine analogs are important as they act by causing depletion of polyamines, due to excessive acetylation and excretion of intracellular amines, followed by cytostasis, apoptosis and cell death (Bacchi et al., 2001). In sporoblasts of *E. cuniculi*, the chitin deacetylase activity (EcCDA) is present in the inner part of the cell wall tightly associated with the plasma membrane. This location was analyzed by biochemical and sequence data, and suggests a role in cell-wall formation. Late sporogony was characterized by the thickening of the chitin-containing endospore, but the mechanism of chitin deposition is unknown. Since the EcCDA enzyme is regularly distributed at the plasma membrane throughout sporogony, chitin may be produced all around the cell (Brosson et al., 2005). The chitin is potent target of nikkomycins due to their deacetylation activity (Bacchi et al., 2001). The fluoroquinolones interact with *E. cuniculi* targets significantly with DNA topoisomerases, which are the enzymes that have evolved to solve the topological problems associated with DNA metabolisms such as transcription, replication, packing and unpacking of DNA in the cell (Hooper, 2000). So, present research studies are concentrating on compounds that target microsporidian polyamines (e.g., polyamine analogs), Chitin (e.g., nikkomycins), and DNA topoisomerases (e.g., fluoroquinolones; Bacchi et al., 2001; Zhang et al., 2005; Didier and Weiss, 2006).

## Entamoeba histolytica

*E. histolytica* is the causative agent of amoebiasis, a contagious disease of the human gastrointestinal tract (Tengku and Norhayati, 2011; Watanabe et al., 2011). It is an organism implicated in both diarrheal disease and invasive disease such as liver abscesses. Metronidazole (**14**) is the first line medication used against the infection but long-term uses produce several side effects in patients (Ordaz-Pichardo et al., 2005). It is potentially carcinogenic to humans because it is genotoxic to human cells (Bendesky et al., 2002). Furthermore, resistance of *E. histolytica* to standard drug metronidazole and relapses of intestinal and hepatic amoebiasis have been reported (Becker et al., 2011; Hwang et al., 2011). The authors have earlier reported diverse functionalized organic compounds synthesized and screened against *E. histolytica* (Azam and Agarwal, 2007, 2015). Nitroimidazole compounds have been used to treat a number of anaerobic bacteria and pathogenic protozoan infections, including *Trichomonas vaginalis, E. histolytica*, and *G. lamblia* since decades (Müller, 1983; Upcroft et al., 1999). Presently, metronidazole, tinidazole (**15**) and ornidazole (**16**) are highly recommended drugs for the treatment of protozoal infections (Azam and Agarwal, 2007). The interesting fact about the metronidazole analogs as an alternative to the treatment of parasitic infections is facilitated by the fact that not only the metronidazole is an effective drug but it also has a side chain which provides an opportunity to carry out various modifications whose significant activity has been reported (Kucik et al., 2004).

Chalcones are the important class of antamoebic drugs and their analogs have been synthesized having better activity. A series of chalcones were synthesized bearing N-substituted ethanamine by aldol condensation reaction and the compound (**17**) was found to be very potent amoebicidal indicating that such compounds can be the effective therapeutic candidates (Zaidi et al., 2015). Metronidazole hydrazone conjugates were synthesized and screened *in vitro* for antiamoebic activity, the compound (**18**) was found more active (Ansari et al., 2015). A series of chloroquinoline-acetamide hybrids were synthesized and the compound (**19**) inhibit the growth of *E. histolytica* (Inam et al., 2015). The *N*-acylhydrazones derived from 7-chloro-4-piperazin-1-yl-quinoline were evaluated against *E. histolytica* and the compound (**20**) was more potent than metronidazole (Inam et al., 2014). Metronidazole-triazole hybrids (**21**) showed amoebicidal activity (Negi et al., 2014). Coordination complexes do exhibit the anti-amoebic activity and the efficacy depends on the selection of both central metal atom and ligands. The terpyridine ligand complexed with divalent metals viz Cu, Co, Mn, Ni, and Zn yield the coordination complexes of general formula [Metal(Fctpy)_2_][PF6]_2_. The complex Ni(Fctpy)_2_][PF6]_2_ (**22**) has been found to be having most promising activity against *E. histolytica* and therefore, can be used as significant drug candidates for amoebiasis (Juneja et al., 2014). Hydrazone and oxadiazoline derivatives of 2-methyl-5-nitro-1H-imidazole were being synthesized and evaluated *in vitro* against HM1:IMSS strain of *E. histolytica* and it was observed that compounds (**23**, **24**) were more potent against amoebiasis (Wani et al., 2013). A series of pyrazoline derivatives were synthesized and their *in vitro* screening against HM1: IMSS strain of *E. histolytica*, results showed the compounds (2E)-1,3-bis(4-methylphenyl)prop-2-en-1-one (**25)** and 1-(4,5-dihydro-5-(4-methoxyphenyl)-3-phenylpyrazol-1-yl)- 2-(5-(4-methoxyphenyl)-1H-tetrazol-1-yl)ethanone (**26**) exhibited the excellent antiamoebic activity and were having the IC_50_-values of 4.19 and 1.16 μM, respectively (Wani et al., 2012b). Compounds bearing a tetrazole and triazine ring motifs in conjugation with a sulphonamide group were synthesized and N, N^∕^-6-(1,3-benzodioxol-5-yl)-(1,3,5-triazine-2,4-diyl)-bis-4-nitrobenzene sulphonamide (**27**) was found to be more potent antiamoebic in nature and was having IC_50_-value of 1.02 μM (Wani et al., 2012a). The *in vitro* antiamoebic activity of 4,6 aminopyrimidines and their sulphonamide derivatives was investigated against *E. histolytica* and it was found that the compound N-[4-(2-chlorophenyl)-6-phenylpyrimidin-2-yl]-benzenesulphonamide (**28**) having IC_50_-value 0.44 μM was most active (Siddiqui and Azam, 2014). Various derivatives of dioxazoles were synthesized and *in vitro* analysis showed that the compound 5-(4-methoxy-phenyl)-3-(5-nitro-2-thienyl)-1,4,2-dioxazole (**29**) was having IC_50_-value 1.60 μM and inhibited the growth of *E. histolytica* significantly (Irfan et al., 2010). Thiosemicarbazides are the focused moieties for antiamoebic drug synthesis and the attempt was made to synthesize a novel series of 4-substituted 1-{[4-(10,15,20-triphenylporphyrin-5-yl)phenyl]methylidene}thiosemicarbazide and their antiamoebic activity was investigated. The 4-(3-methylphenyl)-1-{[4-(10,15,20-triphenylporphyrin-5-yl)phenyl] methylidene}thiosemicarbazide (**30**) with an electron-withdrawing group attached to N(4) exhibited the best antiamoebic activity and was having IC_50_-value 0.538 μM (Bhat et al., 2008). A new series of thiosemicarbazones of 7-hydroxy-8-acetylcoumarin with different thiosemicarbazides were synthesized and were tested against *E. histolytica*. The 7-hydroxy-8-acetylcoumarin-N(4,4)methylbenzyl thiosemicarbazone (**31**) was having IC_50_-value 1.06 μM and found to be antiamoebic (Iqbal et al., 2009). Thiocarbamoyl bis-pyrazoline derivatives were synthesized by cyclization of chalcones with N-4 substituted thiosemicarbazides under basic conditions and the evaluation showed that the compound(4-nitrophenylamino)[5-(4-{1-[(4-nitrophenylamino)thioxomethyl]-3-phenyl(2-pyrazoline-5-yl-phenyl)}-3-phenyl(2-pyrazolinyl)]methane-1-thione (**32**) was having IC_50_-value 0.42 μM and predominantly active as an amoebicidal candidate (Bhat et al., 2009a). An attempt was made to synthesize the amino-5-substituted-(3-phenyl(2-pyrazolinyl))methane-1-thione derivatives, 2-(5-substituted-3-phenyl-2-pyrazolinyl)-1,3-thiazolino[5,4-b] quinoxaline derivatives, and various chalcones derivatives for evaluation against HM1:IMSS strain of *E. histolytica*. The quinoxaline2-{5-[2-(methylethyl)phenyl]-3-phenyl- 2-pyrazolinyl}-1,3-thiazolino[5,4-b]quinoxaline (**33**) showed most promising antiamoebic activity with IC_50_-value of 0.17 μM. The results suggested that the further modification of such compounds can enhance their efficacy (Budakoti et al., 2009). The synthesis of bis-ferrocenyl-substituted core-modified porphyrins derivatives were synthesized under acidic conditions and their *in vitro* screening was performed against HM1:IMSS strain of *E. histolytica.* The promising results showed the1,1^∕^-(10,15-diphenyl-21,23-dithiaporphine-5,20-diyl)bis[2-{[methyl(phenylmethyl)amino]methyl}-ferrocene] (**34**) having IC_50_-value of 0.59 μM was extremely potent (Bhat et al., 2009b). A library of isothiourea derivatives was synthesized and the maximum activity was shown by compound (**35**) with IC_50_-value 2.48 μM. These are the amphiphilic compounds and exist in cationic form which makes them active in biological systems (Kazimierczuk et al., 2010). Benzimidazoles proved to be having antiparasitic activity and 5(6)-chloro-1H-benzimidazole-2-(3H)-thione (**36**) was having IC_50_-value of 0.005 μM for *E. histolytica* which indicates that these derivatives are having higher activity than metronidazole (Valdez et al., 2002). A series of ethyl and methyl quinoxaline-7-carboxylate 1,4-di-*N*-oxide derivatives were synthesized by Beirut reaction and evaluated against HM1:IMSS strain of *E. histolytica*. The methyl 2-acetyl-3-methyl-quinoxaline-7-carboxylate 1,4-di-N-oxide (**37)** and ethyl 2-amide-3-methylquinoxaline-7-carboxylate 1,4-di-N-oxide **(38**) were having IC_50_-value 1.41 and 1.47 μM, respectively and showed higher potency toward amoebicidal activity (Duque-Montaño et al., 2013). The benzologue derivative of nitazoxanide 2-{[(6-Nitro-1, 3-benzothiazol-2-yl)amino]carbonyl}phenyl acetate(**39**) having IC_50_-value 0.297 μM showed better antiamoebic activity as compared to metronidazole and indicated that such derivatives can be explored for the search of effective chemotherapy (Navarrete-Vazquez et al., 2011). The amide derivatives of trifluoromethionine (TFM) have been observed to have excellent activity against *E. histolytica*. Using the TFM as lead 3,4-dimethoxyanilide trifluoromethionine (**40**) with methoxy group at meta and para positions was having IC_50_-value 1.19 μM and was observed most active *in vitro* evaluation (Sato et al., 2010). The compound 1,5-bis[4-(5-methoxy-1H-benzimidazole-2-yl) phenoxy]pentane (**41**) was evaluated *in vitro* against *E. histolytica* showed remarkable IC_50_-value 0.109 μM for amoebicidal potency due to methoxy group attached to benzimidazole ring (Torres-Gomez et al., 2008b). Acetamide and sulfonamide derivatives of imidazole viz N-benzyl-2-(2-methyl-4-nitro-1H-imidazol-1-yl)acetamide (**42)** and 2-methyl-1-[(4-methylphenyl)sulfonyl]-4-nitro-1H-imidazole **(43**) are important class of antiparasitic agents and were having IC_50_-value 3.96 and 3.55 μM, respectively (Hernández-Nunez et al., 2009). Phenyl hydrazine derivatives were synthesized and it was observed that (E)-1-(3-nitrobenzylidene)-2-phenylhydrazine (**44**) was most potent against HM1:IMSS strain of *E. histolytica in vitro* having IC_50_-value 0.84 μM. This indicated that hydrazine derivatives can be explored for the search of effective chemotherapeutic agent for amoebiasis and their mechanism of action needs to be elucidated (Toledano-Magana et al., 2015).

**Figure 1:**
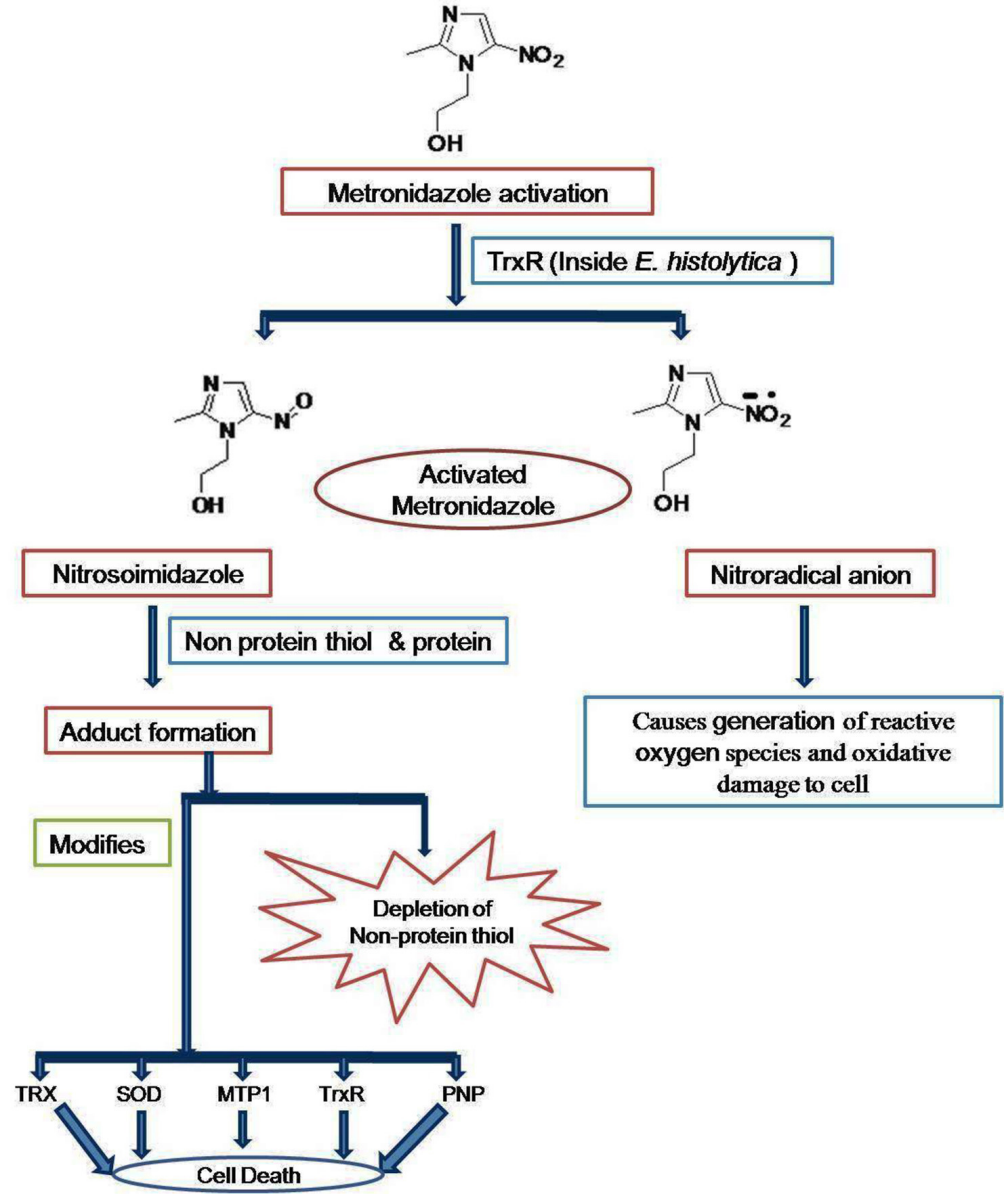
**Mode of action of metronidazole in *E. histolytica***.

Emphasizing the elucidated drug targets and mechanism, the mode of action of metronidazole lies in that it gets reduced to nitroradical anion or nitrosoimidazole by thioredoxin reductase (TrxR) inside *E. histolytica* cell (Leitsch et al., 2007). The nitroradical anion formed reduces O2 and thereby generates reactive oxygen species, which are highly noxious to the cells of microaerophilic *E. histolytica*. However, the nitroso imidazole reacts with non-protein thiols or proteins to forms adduct. The adduct causes depletion of non-protein thiols and the modification of thioredoxin reductase (TrxR), thioredoxin (Trx), superoxide dismutase (SOD), metronidazole target protein 1 (Mtp1), and purine nucleoside phosphorylase (PNP). The combination of proteins with activated metronidazole makes the cells more vulnerable to oxidative stress and thereby kills *E. histolytica* (Figure 1; Samarawickrema et al., 1997; Leitsch et al., 2007). 5-Nitroimidazoles have been the mainstay of treatment, but resistance is a concern and new drug targets are needed. One potential target is the biosynthetic pathway for cysteine which is crucial for growth and various cellular activities (Diamond et al., 1978; Gilin and Diamond, 1980). Besides being precursor for protein biosynthesis, cysteine may compensate for the lack of glutathione, a major component of oxidative stress resistance in many organisms (Fahey et al., 1984; Loftus et al., 2005). It is also needed in the attachment to matrix, elongation and mobility of *E. histolytica* cells (Gilin and Diamond, 1980). In this organism, the condensation of O- acetylserine with sulfide is the major route of cysteine biosynthesis, which involves two key enzymes: O-acetyl-L-serine sulfhydrylase (OASS) and serine acetyltransferase (SATase; Nozaki et al., 1998). In general, in plants and most known organisms, the cysteine biosynthesis pathway is regulated both by the interaction of SAT and OASS to form the cysteine synthase complex (CSC) under sulfur sufficient condition but this type of regulation is absent in *E. histolytica.* These enzymes have been demonstrated to play a central role in controlling the intracellular cysteine concentration in *E. Histolytica* (Nozaki et al., 1999) and block of cysteine biosynthesis is therefore a possible strategy for inhibiting growth of *E. histolytica* (Ali and Nozaki, 2007). *E. histolytica* along with a number of other parasitic protists utilizes an unusual form of phosphofructo-1-kinase (PFK; EC 2.7.1.90) in a central step in carbohydrate metabolism. A homology model of *E. histolytica* PPi-PFK was constructed and screened with various bisphosphonates, which are analogs of pyrophosphate with a carbon instead of oxygen atom and synthetic pyrophosphate. It was found that the Compounds BA49280E (**45**) and CGP42446A (**46**) were good inhibitors of PFK in *E. histolytica.* Based on this study it has been suggested that PFK is attractive target for antiamoebic drugs (Reeves et al., 1974, 1976). Triosephosphate isomerase is another relatively well-studied glycolytic enzyme with both its sequence and structure known in a large number of organisms including *E. histolytica* (Rodriguez-Romero et al., 2002). It has been proposed to be a target for drug design due to presence of a cysteine residue (Cys 14) at the dimer interface in *E. histolytica* (Gómez-Puyou et al., 1995). Alcohol dehydrogenase 2 (EhADH2) is a bifunctionl 97 kDa polypeptide having the alcohol and aldehyde (ALDH) dehydrogenase activities utilize NAD and Fe^2+^ as cofactor (Bruchhaus and Tannich, 1994). This enzyme does not have a homolog in man. However, *E. histolytica* possesses other NADP dependent ADH and ALDH enzymes that could serve a similar function i.e., EhADH1, EhADH3 and EhALDH1. EhADH1 enzyme does not utilize acetyl CoA as substrate thereby suggesting that EhADH2 is solely responsible for the conversion of acetyl CoA to acetaldehyde (Zhang et al., 1994). Based on this data it has been suggested that EhADH2 could also serve as a target for antiamoebic drugs. Calcium is an important secondary messenger in many signal transduction pathways, is thought to be involved in the pathogenesis of amoebiasis and the prevention of the influx of Ca^2+^ can be the potent target (Meza, 2000). In *E. histolytica*, a number of calcium binding proteins have been identified. More importantly two distinct calcium binding proteins EhCaBP1 and EhCaBP2, have been characterized and their role has been simultaneously approached (Sahoo et al., 2003). It has been characterized that cyclosporine A inhibits calcineurin and P-glycoprotein activity and thereby inhibits proliferation of *E. histolytica* (Carrero et al., 2004). Recently it has been shown that when expression of EhCaBP1(a calmodulin containing 134 amino acid long proteins demonstrated to be essential for *Entamoeba*) is blocked, the cellular proliferation in *E. histolytica* gets inhibited (Sahoo et al., 2004).

## Giardia lamblia

*G. lamblia*, an amitochondriate parasite is the major causative agent of human diarrheal disease, infecting an estimated 10% of the world's population both endemically and epidemically (Huang and White, 2006). For the treatment of the intestinal infections caused by *G. lamblia* at least six different classes of drugs are used, but the drugs containing 5-nitroimidazole moiety are most often prescribed due to their significant efficacy. However, the other classes are used when the former fails in treatment. Metronidazole (**14**), tinidazole (**15**), ornidazole (**16**) and secnidazole (**47**) are the 5-nitroimidazole moiety containing drugs. Furazolidone (**48**) a nitrofuran derivative, albendazole (**2**) and mebendazole (**49**) the benzimidazole derivatives, quinacrine (**50**) an acridine derivative, paromomycin (**51**) an aminoglycoside derivative and nitazoxanide (**52**) a 5-nitrothiazolyl derivative are also used for the treatment of *G. lamblia* diarrheal disease depending on the age of patient. But these drugs have various side effects (Reynoldson et al., 1992; Escobedo and Cimerman, 2007). The *in vivo* effects of benzyl and cyclohexyl derivatives like, 2-(1*H*-1-imidazolyl)-1-phenyl-1-ethanol, 2-(2–methyl–1 *H*-1-imidazolyl)–1-phenyl-1-ethanol, 2-(2-methyl-4-nitro-1*H*-1-imidazolyl)-1-phenyl-1-ethanol, 2-(1*H*-1-imidazolyl)-1-cyclohexanol and 1[bis-4-methoxyphenyl-phenylmethyl]-2-methyl-4-nitroimidazole (**53–57**) were studied on the *G. lamblia* trophozoite in the white Syrian mice model and were found to be active (Motazedian et al., 2014). The thieno[2,3-b]pyridine derivatives were synthesized and the 4-(4-methoxyphenylamino)thieno[2,3-b]pyridine-5-carbonitrile having the –OCH_3_ group at para position (**58**) showed the excellent giardicidal effect possibly due to the higher electron density concentrated over phenyl ring (Bernardino et al., 2006). A series of novel hybrids of benzimidazole-pentamidine were prepared and 1,5-bis[2-methoxy-4-(5-methyl-1H-benzimidazole-2-yl)phenoxy] pentane (**59**) was found 3- and 9-folds more potent against *G. lamblia* infection than metronidazole and pentamidine, respectively and was having IC_50_-value of 0.372 μM (Torres-Gomez et al., 2008a). The 2-(trifluoromethyl)benzimidazole derivatives like 5-chloro-1-methyl-2-(trifluoromethyl)benzimidazole (**60**), differently substituted at the 1-, 5-, and 6-positions proved to be active against *G. lamblia* in their *in vitro* analysis and IC_50_-value of 0.042 μM was observed for compound (**60**) (Gabriel Navarrete-Vaa Zquez et al., 2001). A library of chalcones was prepared by condensing substituted acetophenones with benzaldehydes using the Claisen–Schmidt base-catalyzed aldol condensation reaction. Among these chalcones, substituted with –OCH_3_, -H, -Cl, -F at different positions (E)-3-(6-flourophenyl)-1-(4-methoxyphenyl)prop-2-en 1-one (**61**) with IC_50_-value of 12.72 μM showed the excellent results toward the giardial infection (Montes-Avila et al., 2009). The benzimidazole derivative 5(6)-chloro-1H-benzimidazole-2-(3H)-thione (**62**) was reported active *in vitro* against *G. lamblia* with IC_50_-value of 0.005 μM and inhibits tubulin polymerization (Valdez et al., 2002). The albendazole analogs 1-methyl-6-(propylthio)-2-(trifluoromethyl)-1H-benzimidazole (**63**) and 5(6)-(propylthio) -2-(trifluoromethyl)-1H-benzimidazole (**64**) were having IC_50_-values 1.403 and 1.515 μM, respectively are active toward giardiasis. The mebendazole analogs 5-benzoyl-1-methyl-2-(trifluoromethyl)-1H-benzimidazole (**65**) and 6-benzoyl-1-methyl-2-(trifluoromethyl)-1H-benzimidazole (**66**) have also been found to be active against *G. lamblia* and these were having IC_50_-values 1.098 μM 1.285 μM, respectively (Navarrete-Vazquez et al., 2003). Among the series of 3-tetrazolylmethyl-4H-chromen-4-ones the compound 3-((1-cyclohexyl-1H-tetrazol-5-yl)((3,4,5-trimethoxyphenyl)amino)methyl)-4H-chromen-4-one (**67**) having IC_50_-value of 171.4 μM can represent the suitable alternatives against resistant *G. lamblia* parasites(Cano et al., 2014). Thiazole derivatives were synthesized and screened against *G. intestinalis*, two novel methyl 5-nitro-1,3-thiazol-2-ylcarbamate (**68**) and ethyl [(5-nitro-1,3-thiazol-2-yl)amino](oxo)acetate (**69**) proved to be potent giardicidal and were having IC_50_-value of 0.010 and 6.410 μM, respectively(Nava-Zuazo et al., 2014). By using the Ugi-azide multicomponent reaction, novel 3-tetrazolylmethyl-4H-chromen-4-ones were synthesized and evaluated against *G. lamblia*. The *in vivo* investigation demonstrated that these compounds like (**70**) could be specifically considered drug candidates for giardial infection (Cano et al., 2014). A series of novel hybrids from benzimidazole and pentamidine were prepared and tested *in vitro* against the protozoa and it was observed that the compound1,5-bis[2-methoxy-4-(5-methyl-1H-benzimidazole-2- yl)phenoxy] pentane (**71**) with IC_50_-value 0.372 μM showed 3- and 9-fold more activity against *G. lamblia* than metronidazole and pentamidine, respectively (Torres-Gomez et al., 2008b). Benzologues of nitazoxanide and tizoxanide were synthesized and tested *in vitro* against *G. intestinalis.* The 2-{[(6-nitro-1,3-benzothiazol-2-yl)amino]carbonyl}phenyl acetate (**39**) was having IC_50_-value 3.515 μM and was18-times more potent than metronidazole (Navarrete-Vazquez et al., 2011). Acetamide derivatives of imidazole like N-(4-cyanophenyl)-2-(2-methyl-4-nitro-1H-imidazol-1-yl) acetamide (**72**) and 2-methyl-4-nitro-1-[(4-nitrophenyl)sulfonyl]-1H-imidazole (**73**) were screened *in vitro* and were found active against *G. lamblia* with IC_50_-value 11.25 and 7.50 μM, respectively. These can be further modified to enhance their activity (Hernández-Nunez et al., 2009).

**Figure 2:**
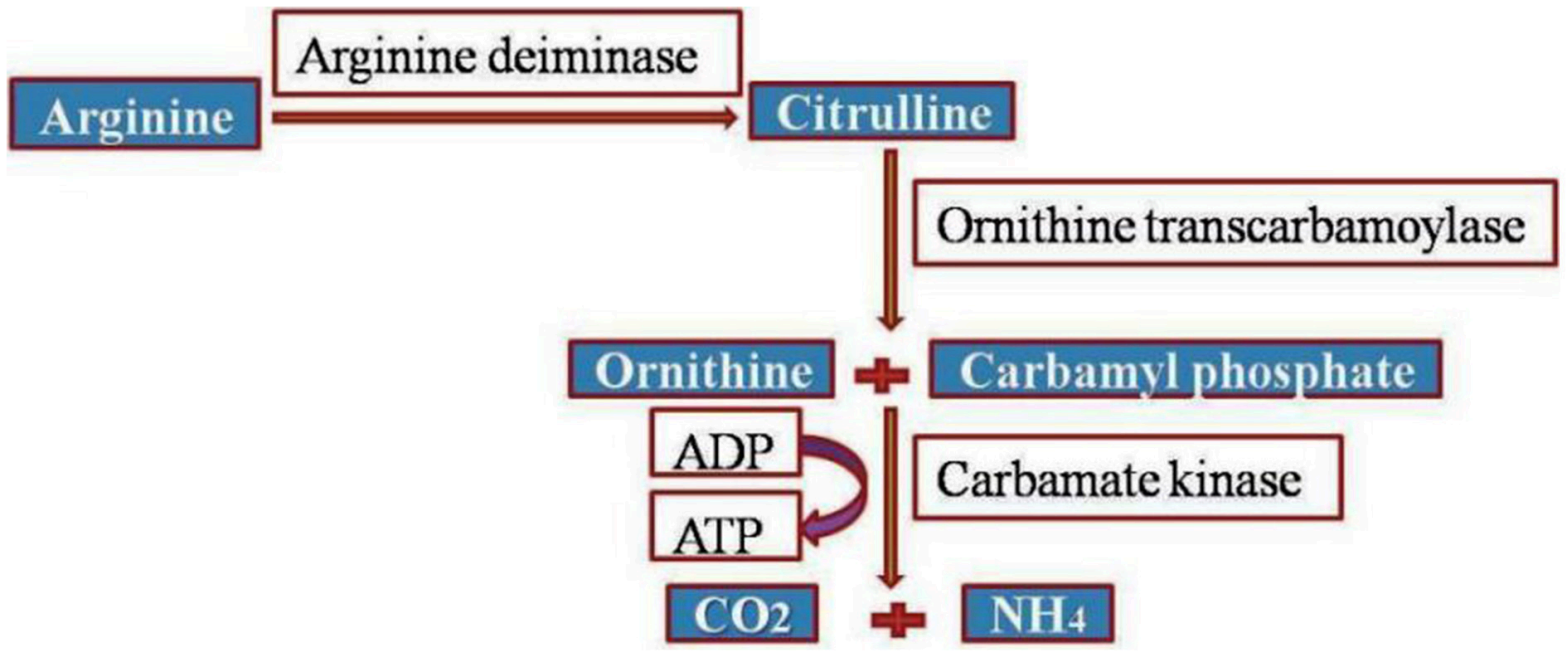
**Arginine dihydrolase pathway in *G. lamblia***.

**Figure 3:**
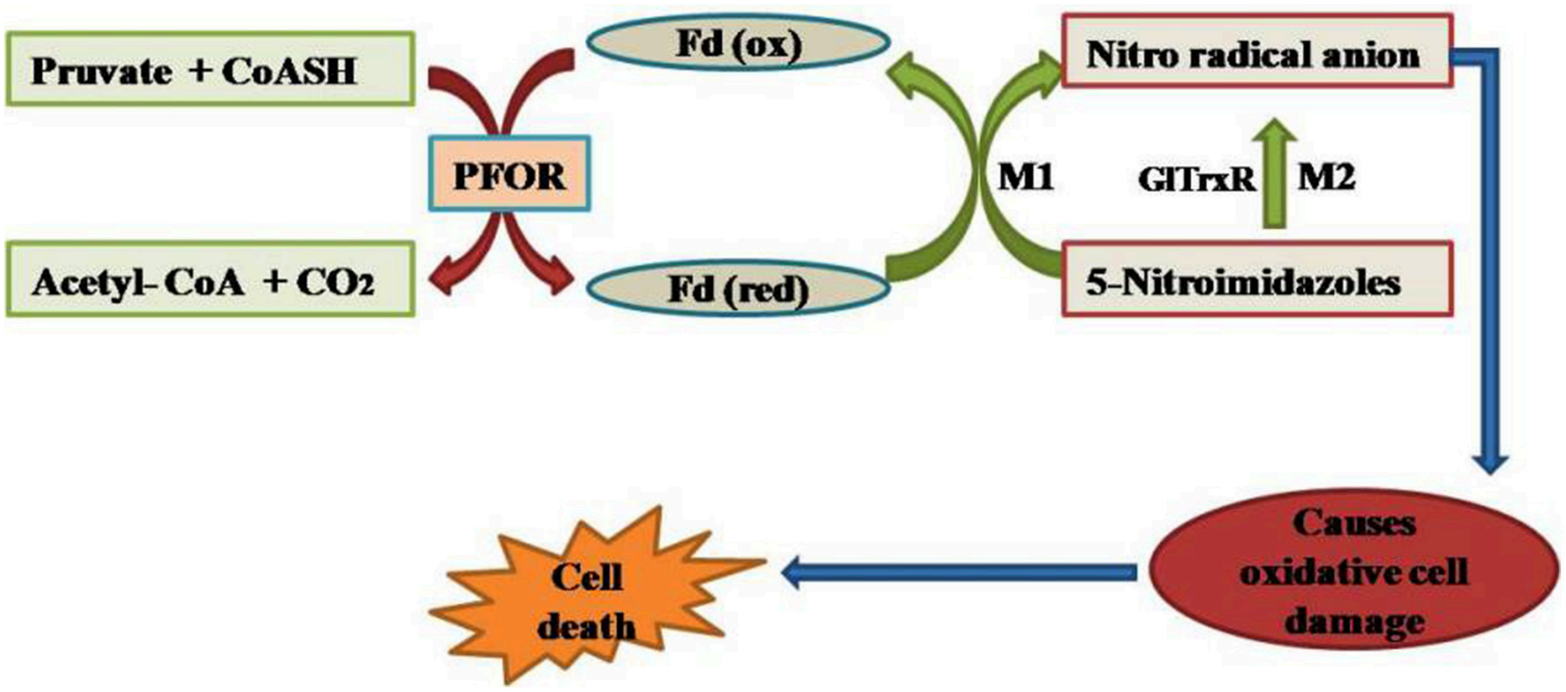
**Mode of action of nitroimidazoles in *G. lamblia***.

Accentuating the targets, *G. lamblia* utilizes the arginine dihydrolase pathway to produce ATP from ADP and L-arginine (Schofield et al., 1990), a pathway which is lacking in higher eukaryotes including humans. The arginine dihydrolase pathway employs three enzymes, arginine deiminase, ornithine transcarbamoylase, and carbamate kinase (CK; EC 2.7.2.2). CK catalyzes the last step of the pathway, converting carbamoyl phosphate and ADP into carbamate and ATP (Figure 2; Lim et al., 2013). Carbamate kinase is essential enzyme of *Giardia lamblia*, and the antialcoholism drug disulfiram (**74**) kills the trophozoites and inhibits this enzyme. Disulfiram and their analogs act by modifying Cys242 adjacent to the active site and cure of giardiasis in mice has also been reported (Galkin et al., 2014). Nitazoxanide, nitroimidazoles, metronidazole and tinidazole use nitroreductases such as pyruvate ferredoxin oxidoreductase (PFOR) as their target sites in Giardial infection (Hoffman et al., 2007; Müller et al., 2007). The 5-nitroimidazoles like metronidazole act in *G. lamblia* by two distinct mechanisms. In one mechanism metronidazole gets reduced to toxic nitro-radical anion via pyruvate ferredoxin oxidoreductase/ferredoxin (PFOR/Fd) couple in presence of pyruvate and the cofactor CoASH during the electron transport pathway in *G. lamblia.* In the second mechanism metronidazole reduction occurs due to the activity of *G. lamblia* thioredoxin reductase (GlTrxR). The radicals formed cause oxidative damage to *G. lamblia* cell and thereby kills parasite (Figure 3).

Protein disulfide isomerases, specifically PDI2 and PDI4 have recently been reported as promising targets for new drugs effective against giardiasis (Müller et al., 2007). Furazolidone gets reduced by NADH oxidase when the reduced products obtained disrupt the action of DNA in Giardial infection (Brown et al., 1996; Upcroft and Upcroft, 1998). Recently, Hsp90 inhibitors as potential leads for antiparasitic chemotherapy have been reported. Hsp90 inhibitor prodrug (SNX-5422) was evaluated in animal models to proof the principle that an oral drug could be effective (Debnath et al., 2014). Metronidazole analogs were more active than metronidazole in changing significantly the morphology and ultrastructure of the parasite *G. lamblia*. The analogs affected parasite cell vesicle trafficking, autophagy, and triggered differentiation into cysts of *G. lamblia* (Busatti et al., 2013). The novel nitroreductase target sites viz GlNR1 and GlNR2 have been identified to be the potent targets for nitroimidazole, metronidazole, nitrothiazolide and nitazoxanide in *G. lamblia* (Muller et al., 2015).

## Cryptosporidium parvum

The protozoan *C. parvum* causes cryptosporidiosis which is associated with watery diarrhea that may sometimes be profuse and prolonged in children (Current and Garcia, 1991; Bouzid et al., 2013). It is the second most common protozoan pathogen responsible for severe diarrhea and was also associated with death in young children under 12–23 months of age (Kotloff et al., 2013). Currently nitazoxanide (**52**), paromomycin (**51**), and azithromycin are the most commonly used for the treatment of cryptosporidiosis. However, nitazoxanide (**52**) is effective in the immunocompetent and ineffective in the immunocompromised patients (Mead, 2002; Gargala, 2008). The available medication is not effective in all the patients besides having side effects of headache, nausea, stomach pain, severe or persistent dizziness, shortness of breath and unusual tiredness. Progress in the development of anti-cryptosporidial drugs has been very slow due to the difficulty in the *in vitro* culture of *Cryptosporidium* (Abrahamsen et al., 2004; Andrews et al., 2014). Many drug targets are not present in this parasite because it has completely lost the plastid derived apicoplast. Its mitochondrion lacks the citrate cycle and cytochrome based respiratory chain which directs the novel target identification for drug development. Recently a research group synthesized 11 derivatives of benzimidazole, out of which three compounds (**75**–**77**) showed anti-cryptosporidic effect equivalent to paromomycin (Graczyk et al., 2011). The dicationic carbazole compounds are active against *C. parvum* and compound (**78**) was elucidated to be potent against cryptosporidiosis (Blagburn et al., 1998). The macrolide antibiotics like spiramycin (**79**), roxithromycin (**80**), and clarithromycin (**81**) were thought to be promising drugs against cryptosporidiosis but the final clinical trials have not yet been completed. There is the intense need for the development of effective chemotherapy for cryptosporidiosis in immunodeficient patients (Saez-Llorens, 1989; Uip et al., 1998). The nitrogen-containing bisphosphonates (N-BPs) exhibit anticryptosporiudium activities in low micromolar concentrations. It has been observed that NBPs act on the novel non-specific polyprenyl pyrophosphate synthase that can synthesize isoprenoids from C20->C45 in *Cryptosporidium spp* (Artz et al., 2008).

In *C. parvum*, phosphofructokinase enzyme is pyrophosphate specific (PPi-PFK; Denton et al., 1996). The accepted dogma is that PPi-PFK is advantageous to anaerobic organisms because its economic effect on the cell's consumption of ATP means that the net production from glycolysis is three ATP molecules per glucose molecule rather than the more usual two ATP molecules (Coombs and Müller, 1995). The critical role played by PPi-PFK in energy metabolism together with difference from the human host PFK, make it an attractive target for drug. A mannitol cycle is an important part of energy metabolism for *C. parvum*. A key feature of the cycle is that the enzyme initiates mannitol biosynthesis, mannitol-1-phosphate dehydrogenase, is regulated by the binding of a specific inhibitor, a protein that has been found to belong to the 14–3–3 group of proteins. Inhibition of mannitol synthesis would be likely to have a severe impact upon the oocyst stage of the parasite and so reduces transmission (Schmatz, 1997). The inosine-5′-monophosphate dehydrogenase (IMPDH) and proteases are also proposed to be the target sites in cryptosporidial infection (Mandapati et al., 2014). It has a bacterial-type inosine-5′-monophosphate dehydrogenase (IMPDH) as the only means to convert AMP to GMP (Umejiego et al., 2008). The target site of the paromomycin is the A site of ribosome where upon action it disrupts the protein biosynthesis in *C. parvum* (Müller et al., 2007). Acyl-coenzyme-A synthetases have been observed to be inhibited by triacsin C in cryptosporidial infection in mice (Guo et al., 2014). The dicationic-substituted aromatic molecules act by binding to the minor groove of DNA in the *Cryptosporidium* and thereby affects. DNA-associated enzymes such as topoisomerases, endo and exonucleases or other processes, such as DNA replication, recombination, and repair (Hildebrandt et al., 1998). Mebendazole or albendazole acts by interacting with the colchicine site of tubuline in the microtubules of *Cryptosporidium* spp (Brown et al., 1996).

## Blastocystis hominis

*Blastocystis hominis* is a unicellular anaerobic protozoon and infects the human gastrointestinal tract. It gets transmitted via animal contact, water and food contaminated with excreted cysts of the organism (Eroglu and Koltas, 2010). The pathogenesis of *B. hominis* leads to symptoms such as diarrhea, flatulence, anorexia, abdominal pain, vomiting, perianal pruritics, and bloating (Sheehan et al., 1986; Chen et al., 2014). The currently used drugs against this infection are metronidazole (**14**) or tinidazole (**15**) (Sohail and Fischer, 2005; Tan, 2008). Metronidazole commonly used for the treatment of blastocystosis induces programmed cell death in the *B. hominis*(Nasirudeen et al., 2004). The reduction of the nitro group cytotoxic radicals in mitochondria causes the death of concerned pathogen (Kulda, 1999). Trimethoprim-sulfamethoxazole (TMP-SMX) (**82**, **83**) is used as the second line medication when the first line treatment by metronidazole (**14**) does not work (Ok et al., 1999). Other compounds being occasionally used include pentamidine (**84**), iodochlorhydroxyquin (**85**), furazolidone (**48**), iodoquinol (**86**), tinidazole (**15**), nitazoxanide (**52**), and emetine (**87**) (Moghaddam et al., 2005; Sohail and Fischer, 2005). Paramomycin (**51**), a broad spectrum aminoglycoside antibiotic has exhibited superior performance in comparison to metronidazole (**14**). Therefore, it can act as the therapeutic agent for *B. hominis* infections (Van Hellemond et al., 2013).

The mechanism of action of several drugs, included metronidazole, have been studied and found linked to the induction of programmed cell death in the parasite, when treated with a surface reactive cytotoxic monoclonal antibody (MAb 1D5). The central vacuole of *B. hominis* grown in normal physiological conditions may contain lipid granules or electron-dense particles. This central vacuole is used for programmed cell death (PCD) process and it acts as a repository where apoptotic bodies are stored before being released into the extracellular space. Central vacuole is potent target for apoptosis (Nasirudeen et al., 2001, 2004).

## Isospora belli

Isosporiasis is human intestinal disease caused by the parasite *I. belli*. It is most commonly observed in Africa, Asia and South America. The currently used drugs against this infection is combination of trimethoprim (**82**) and sulfamethoxazole (**83**) (Goodgame, 2003; Hunter et al., 2004). The pyrimethamine (**88**) and sulfadiazine (**89**) also cure the diarrheal disease caused by *I. Belli* (Ferguson et al., 1980; Ebrahimzadeh and Bottone, 1996). Macrolide antibiotics like sirimamycin proved to be effective when pyrimethamine-sulfadoxine (**88**, **90**) fails to cure the intestinal infection by *I. belli* specifically in AIDS patients (Ferguson et al., 1980). Similarly, roxithromycin (**91**) is also observed to be effective for chronic *I. belli* induced diarrhea when TMP-SMX (**82**, **83**) or pyrimethamine (**88**) treatments become inadequate (Musey et al., 1988). It has been reported that metronidazole (**14)**, tinidazole (**15**), quinacrine (**50**), and furazolidone (**48**) also cure the *I. belli* induced diarrhea but to lesser extent. However, the treatment with metronidazole is more efficient than tinidazole (**15**), quinacrine (**50**), and furazolidone (**48**) (Trier et al., 1974; Syrkis et al., 1975; Butler and De Boer, 1981; Weiss et al., 1988). The antimalarial compounds, primaquine phosphate (**92**) and chloroquine phosphate (**93**) may be helpful in immunocompetent patients with persistent *I. belli* infection (Trier et al., 1974). The synthesis of more potent drugs and tracing the valuable targets is the important area of research for *I. belli* intestinal infection.

## Cyclospora cayetanensis

Cyclosporiasis is the gastrointestinal illness caused by the microscopic parasite *C. cayetanensis*. The extent of illness varies based on age, condition of the host and size of the infectious dose (Goodgame, 2003). Trimethoprim–sulfamethoxazole (**82**, **83**) was at first used to treat cyclosporiasis in immunocompetent and immunocompromised patients (Madico et al., 1993; Guerrant et al., 2001). However, the patients who are allergic to sulpha drugs, the ciprofloxacin (**94**) or trimethoprim (**82**) is prescribed (Verdier et al., 2000). Nitazoxanide (**52**) had no severe side effects and was tolerated effectively and can be better option for intestinal infection caused by *C. cayetanensis* (Diaz et al., 2003). The development of effective drugs and the quest of essential targets are of prime importance to treat cyclosporiasis.

## Conclusion

Drug development for parasite-induced diarrheal disease is in progress. There is a pressing need for the identification of compounds which are efficacious in *in vivo* animal studies and can be subjected to clinical trials. Drug development for *C. parvum* is particularly challenging because of the difficulty of *in vitro* screening. Maximum effort should be directed toward new compounds to treat cryptosporidiosis because of the limited availability of effective drugs. Resistance to the current drugs, metronidazole, paromomycin, and nitazoxanide is a major concern. A major research focus on key biochemical pathways, identification of essential targets, better assay methods, and *in vivo* testing are urgently needed to identify new chemotherapeutic agents for parasite-induced diarrheal disease. As per our contemplated study, there is a need for the determination of mechanism of action of the synthesized compounds against *E. histolytica* and *G. lamblia*. The synthesized heterocyclic scaffolds bearing different substituents that have been found to be active *in vivo* studies against the parasites causing diarrhea, can be further explored as future drug candidates with higher efficacy, resistance effectiveness, and lesser side effects.

The bold numbers in braces refer to the chemical structures of the respective compounds (see Supplementary Material).

### Conflict of interest statement

The authors declare that the research was conducted in the absence of any commercial or financial relationships that could be construed as a potential conflict of interest.
