# Erratum: Parasitic diarrheal disease: drug development and targets

**DOI:** 10.3389/fmicb.2015.01328

**Published:** 2015-11-16

**Authors:** Frontiers Production Office

**Affiliations:** Frontiers Production Office, FrontiersLausanne, Switzerland

**Keywords:** diarrhea, causative parasitic agents, chemotherapy, drug targets, therapeutic developments

Reason for Erratum:

Due to a technical error, the Supplementary Material for the article, containing the chemical structures was omitted from the published article. The publisher apologizes for this error and the correct Supplementary Material has been added to the original article.

This error does not change the scientific conclusions of the article in any way.

