# Supplementary figures and images for: Parasitic diarrheal disease: drug development and targets
Source: Front Microbiol. 2015 Oct 27;6:1183. doi: 10.3389/fmicb.2015.01183 (PMC4621754; doi:10.3389/fmicb.2015.01183)

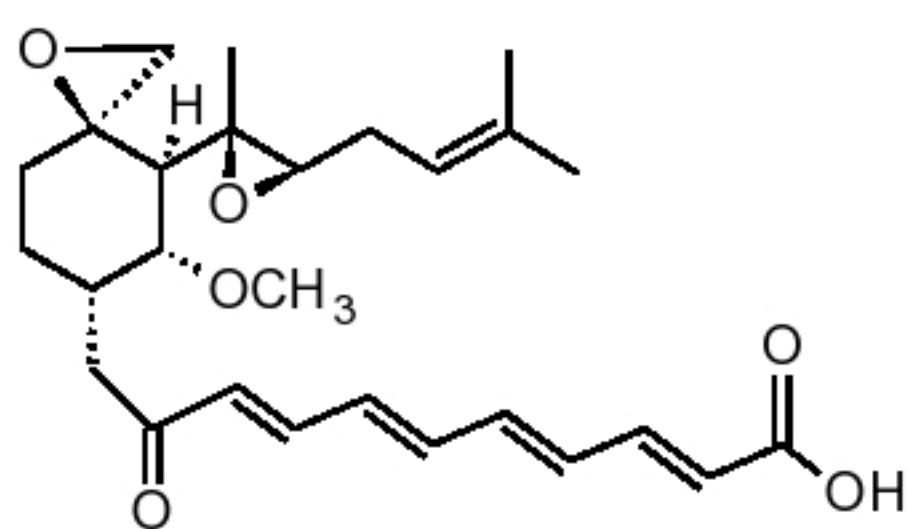

1

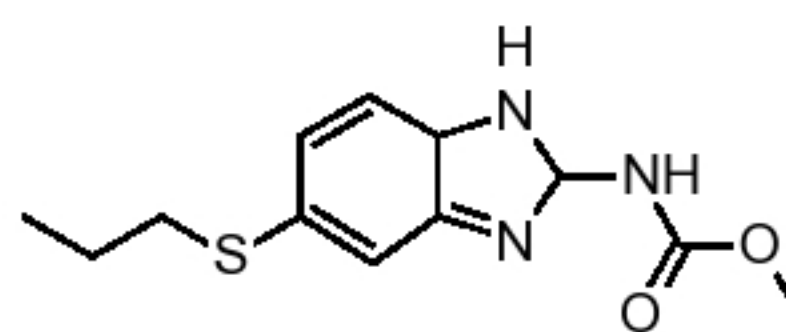

2

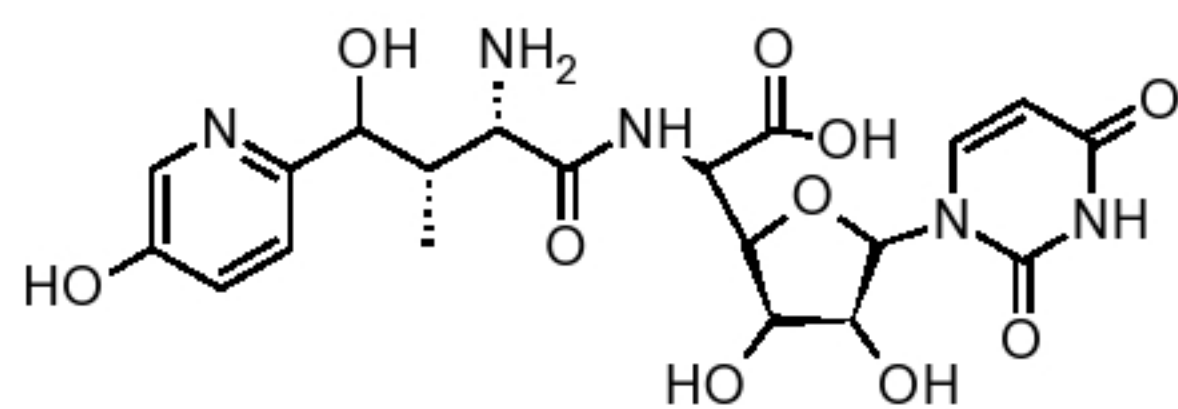

3

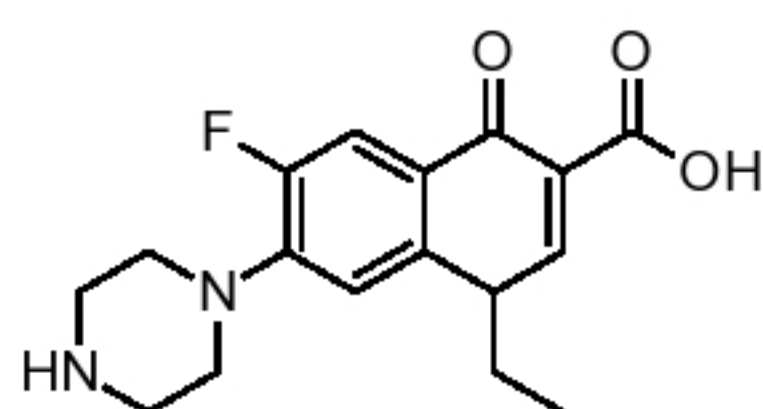

4

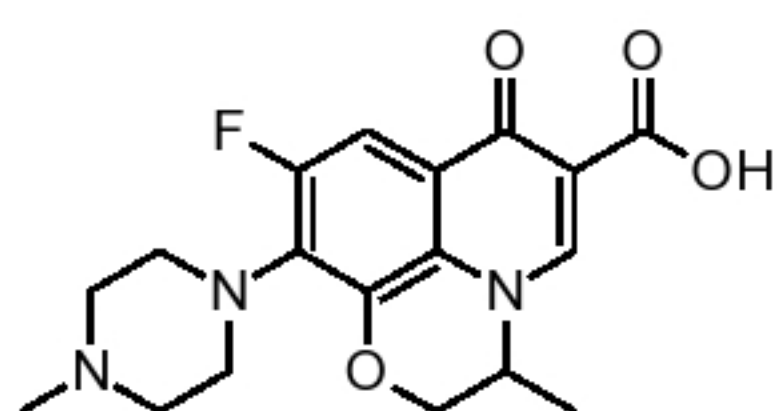

5

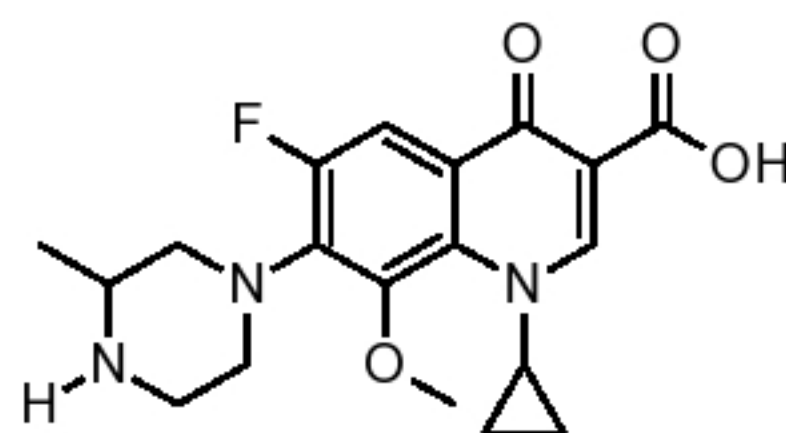

6

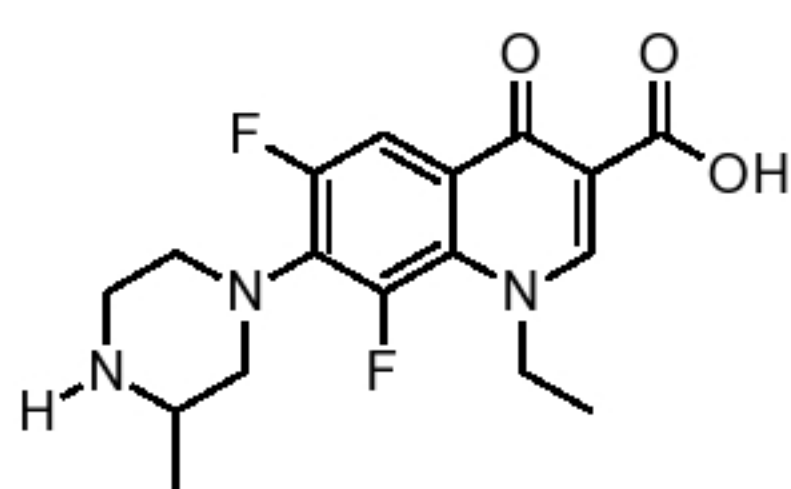

7

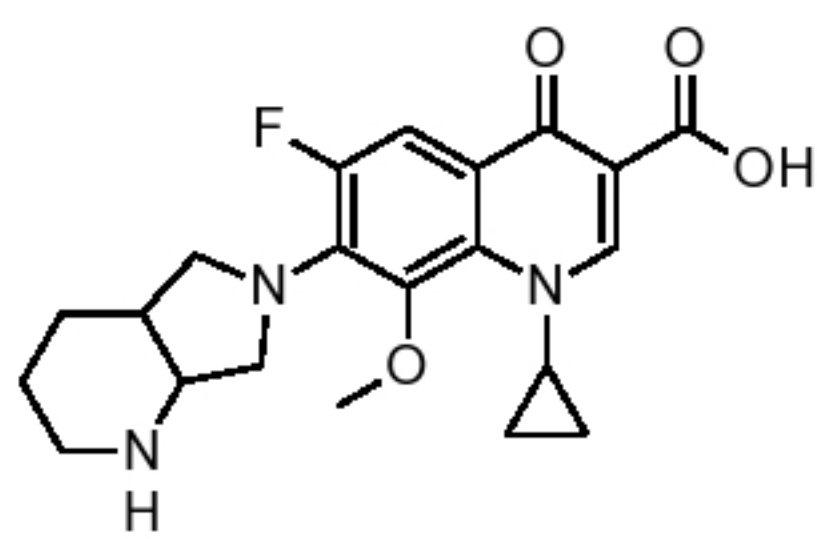

8

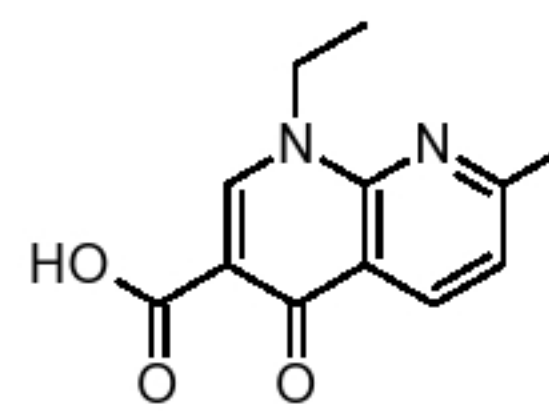

9

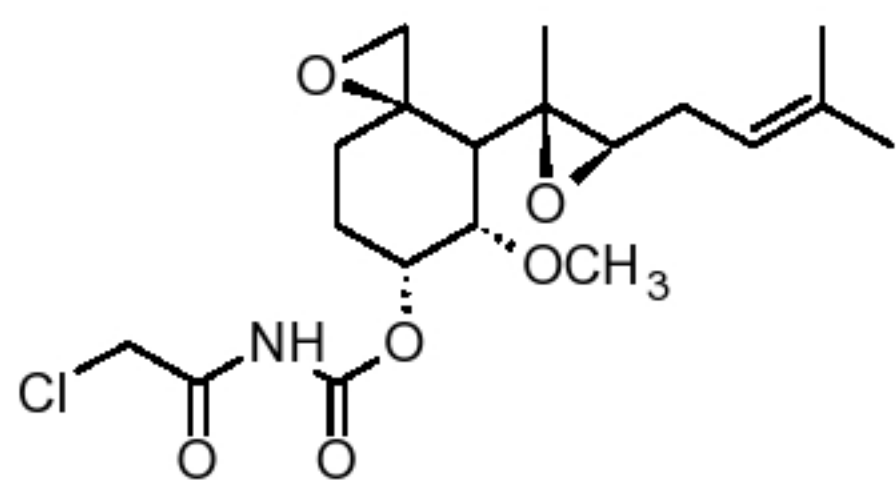

10

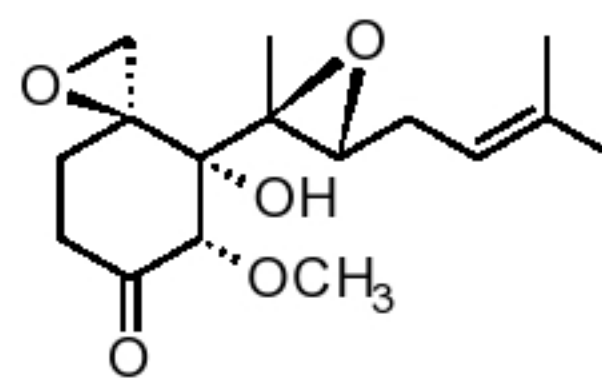

11

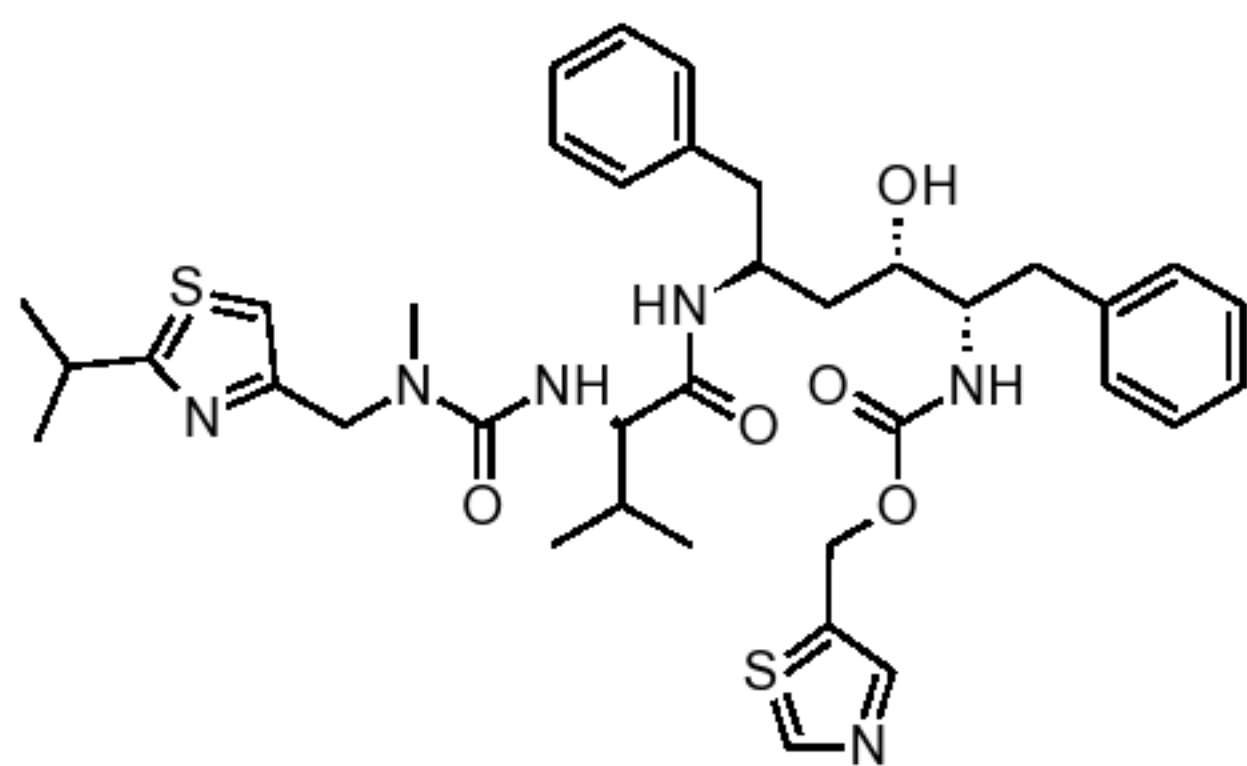

12

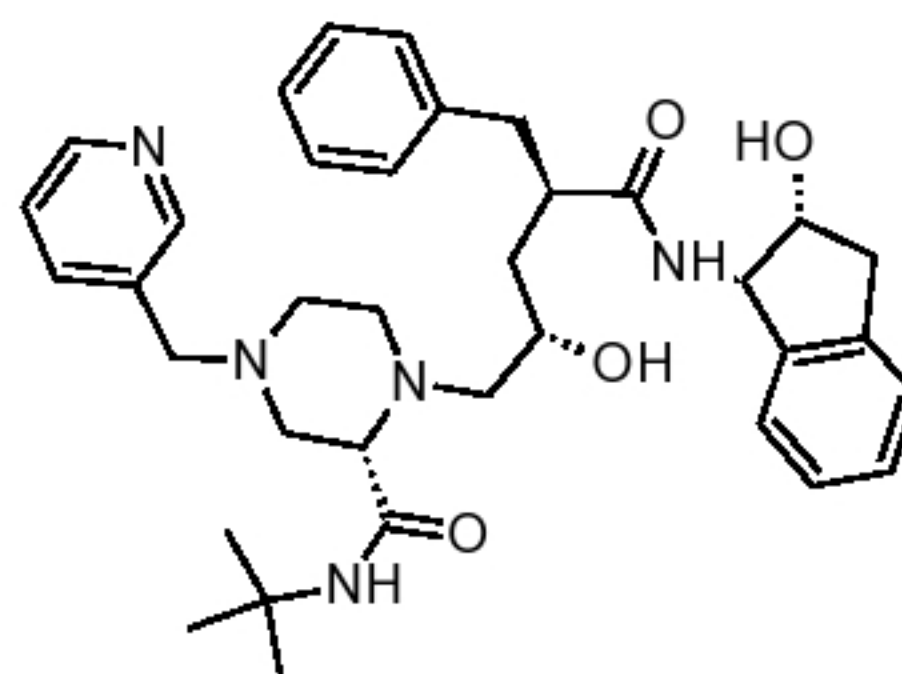

13

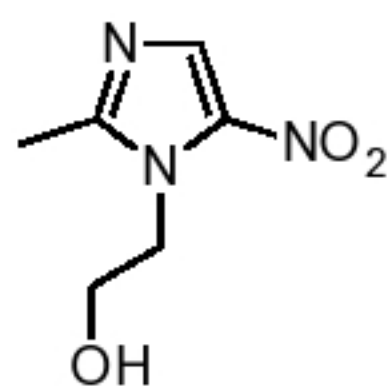

14

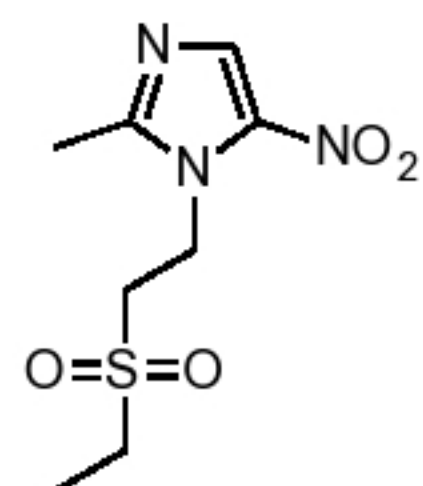

15

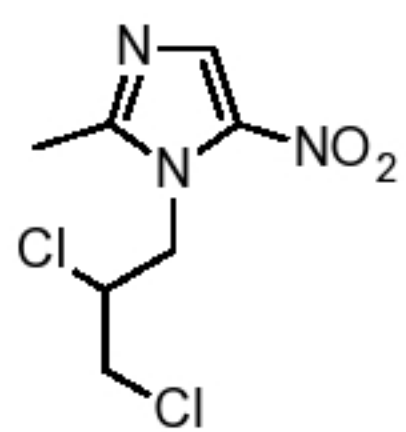

16

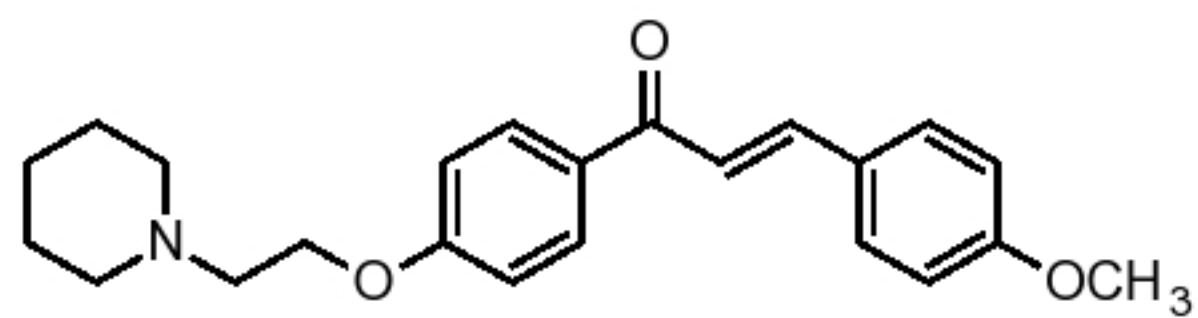

17

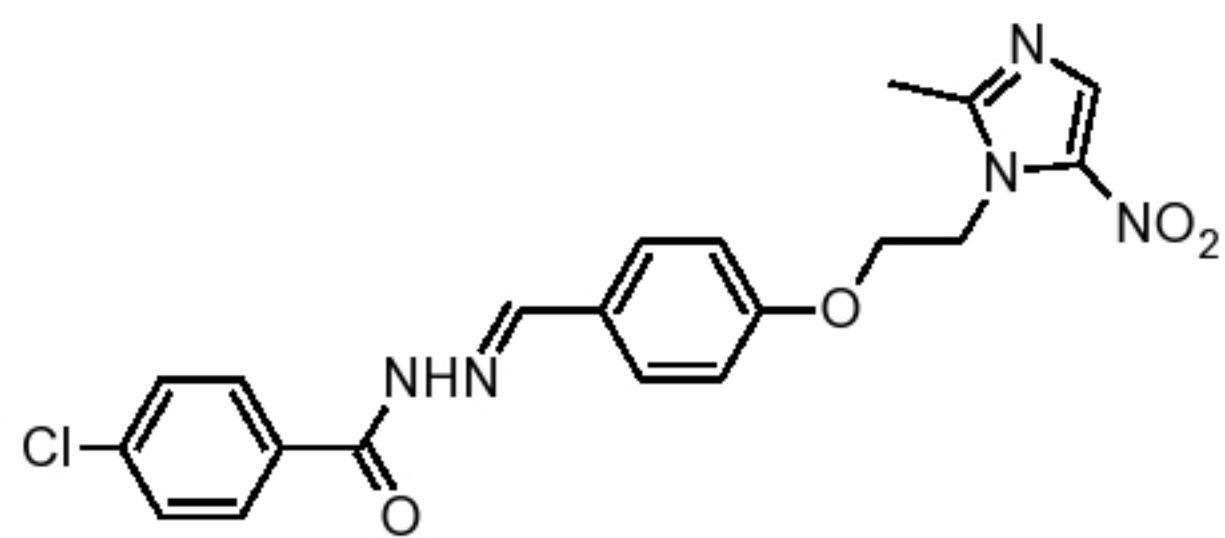

18

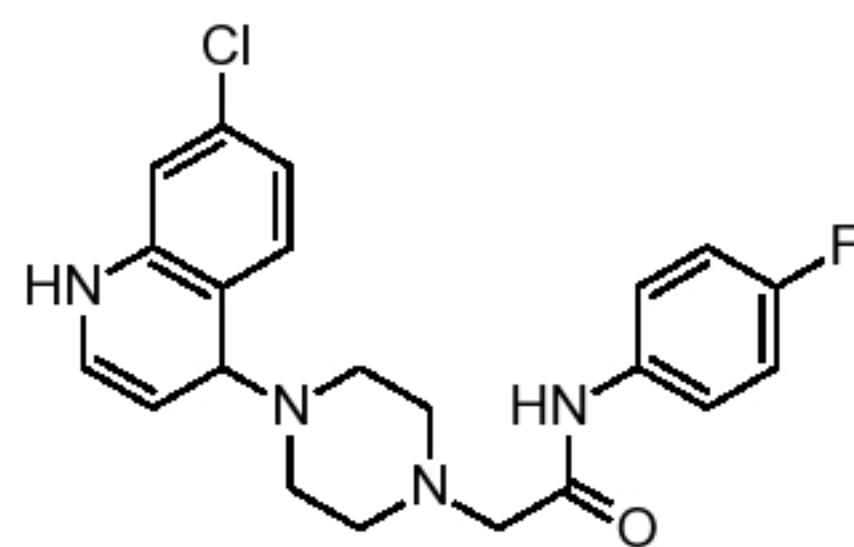

19

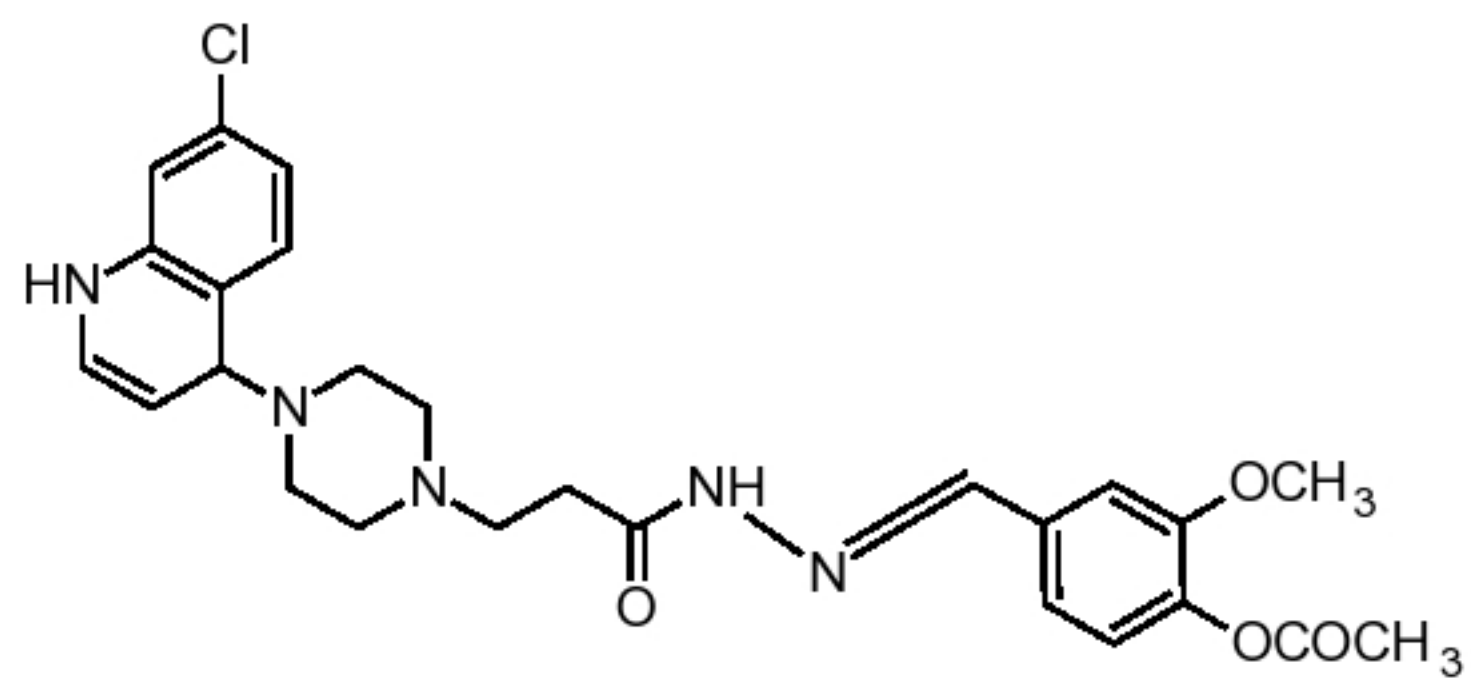

20

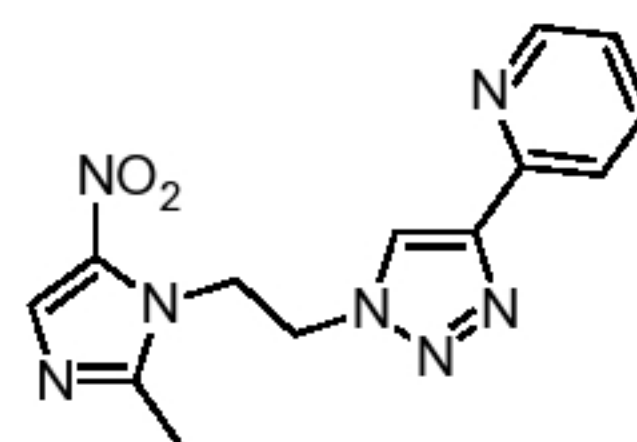

21

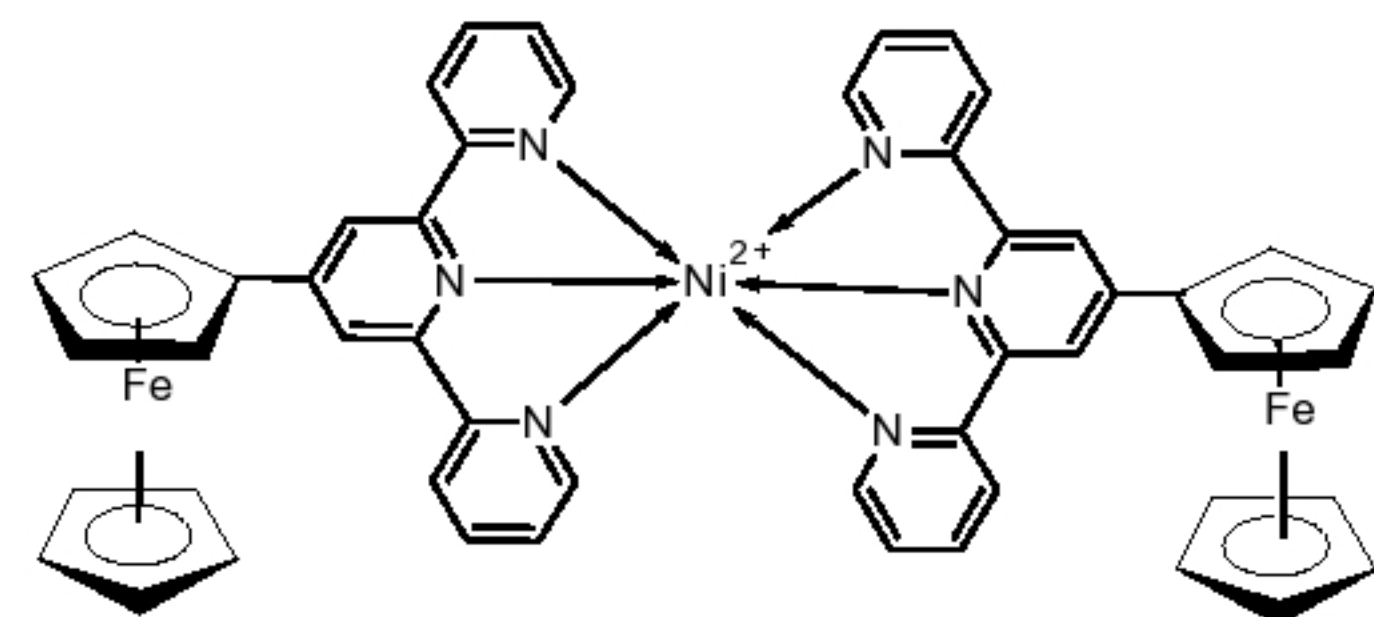

22

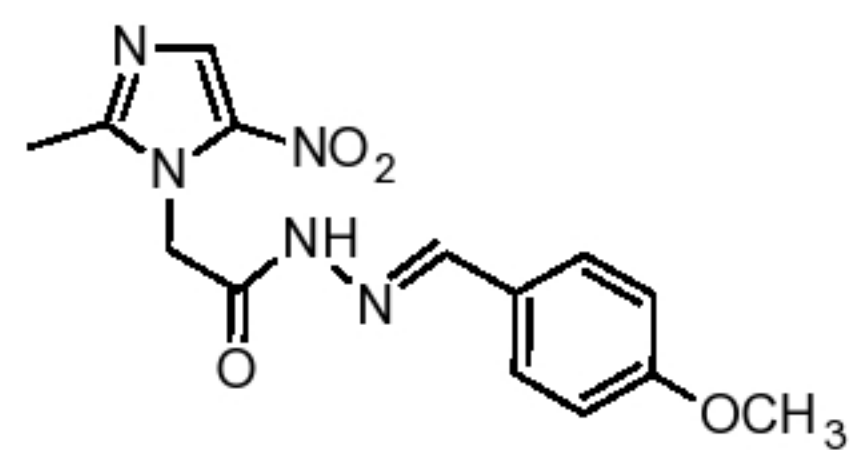

23

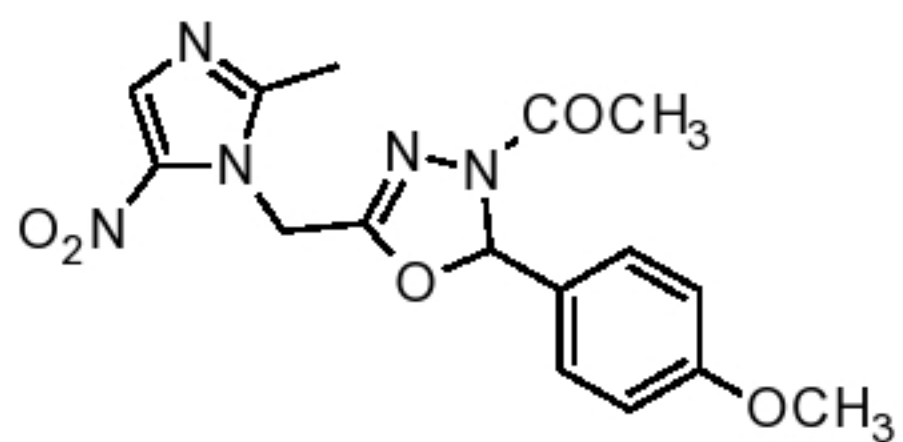

24

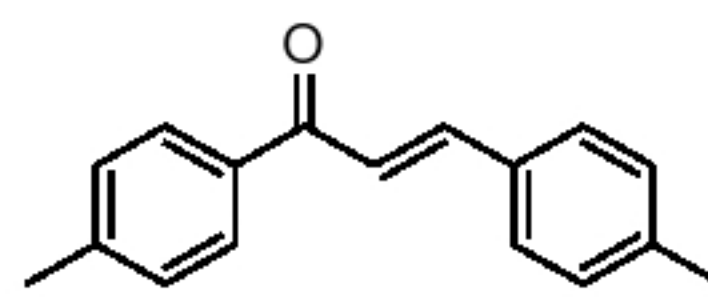

25

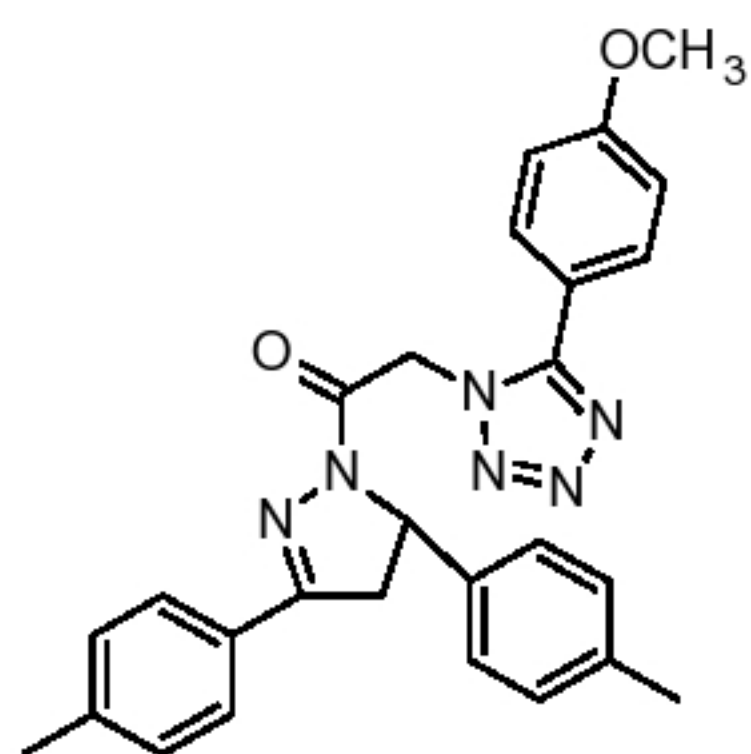

26

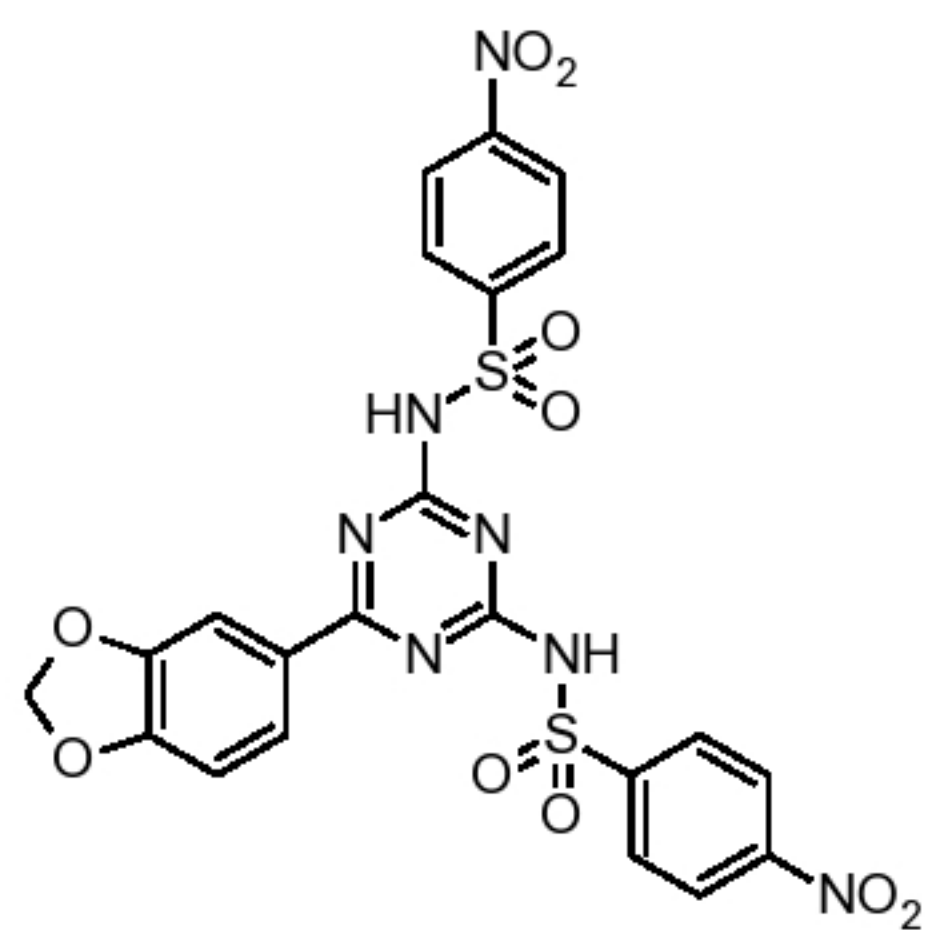

27

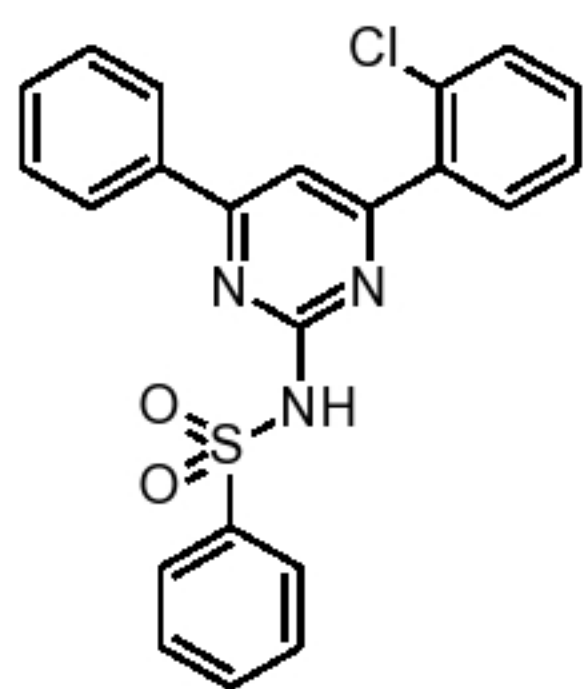

28

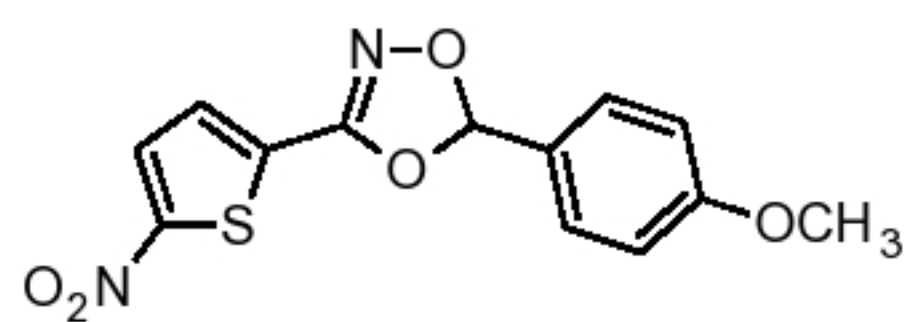

29

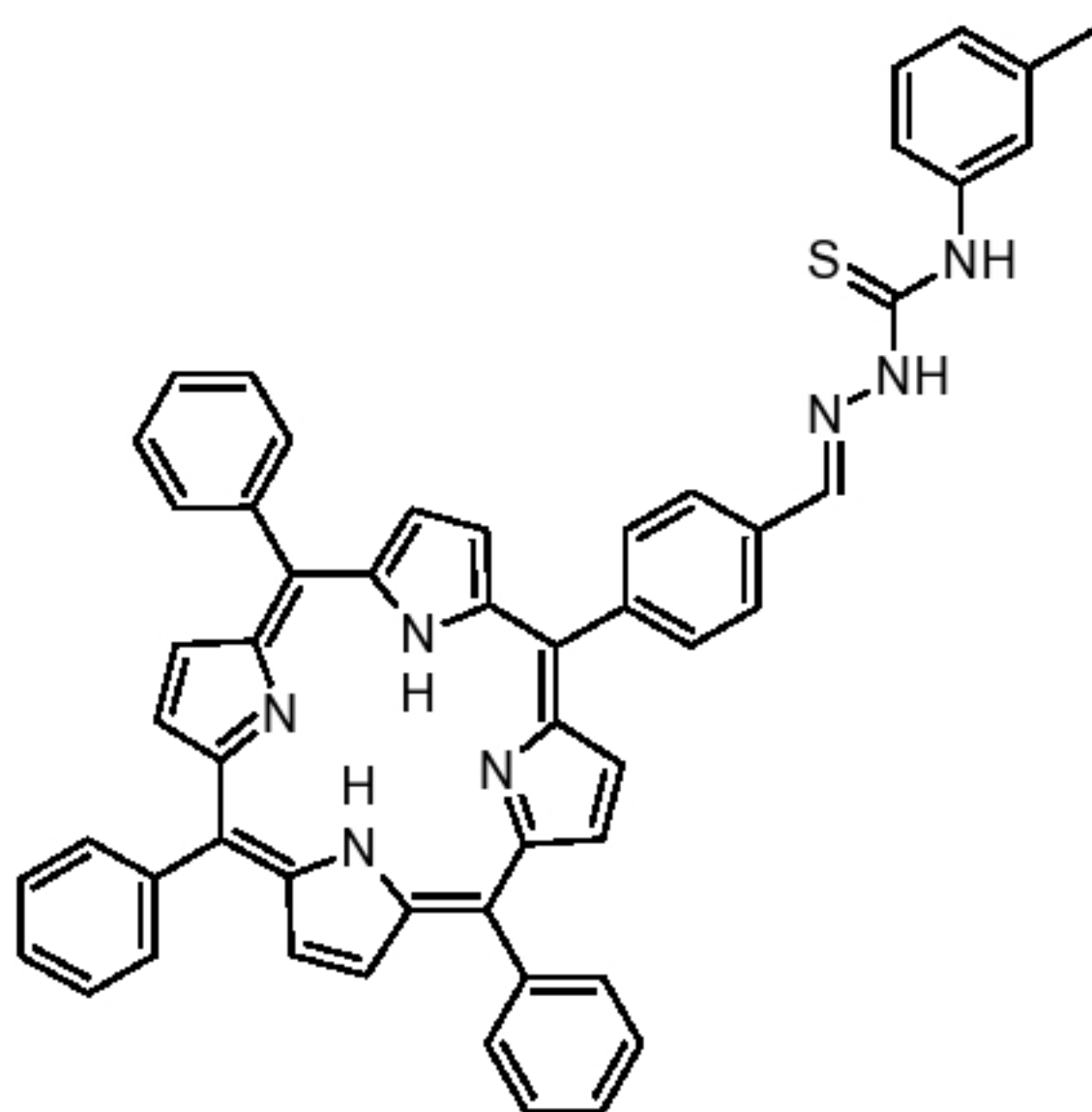

30

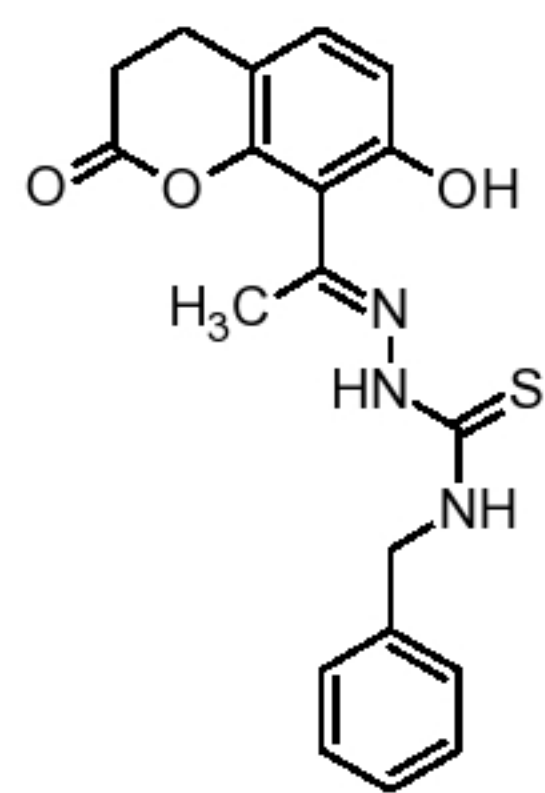

31

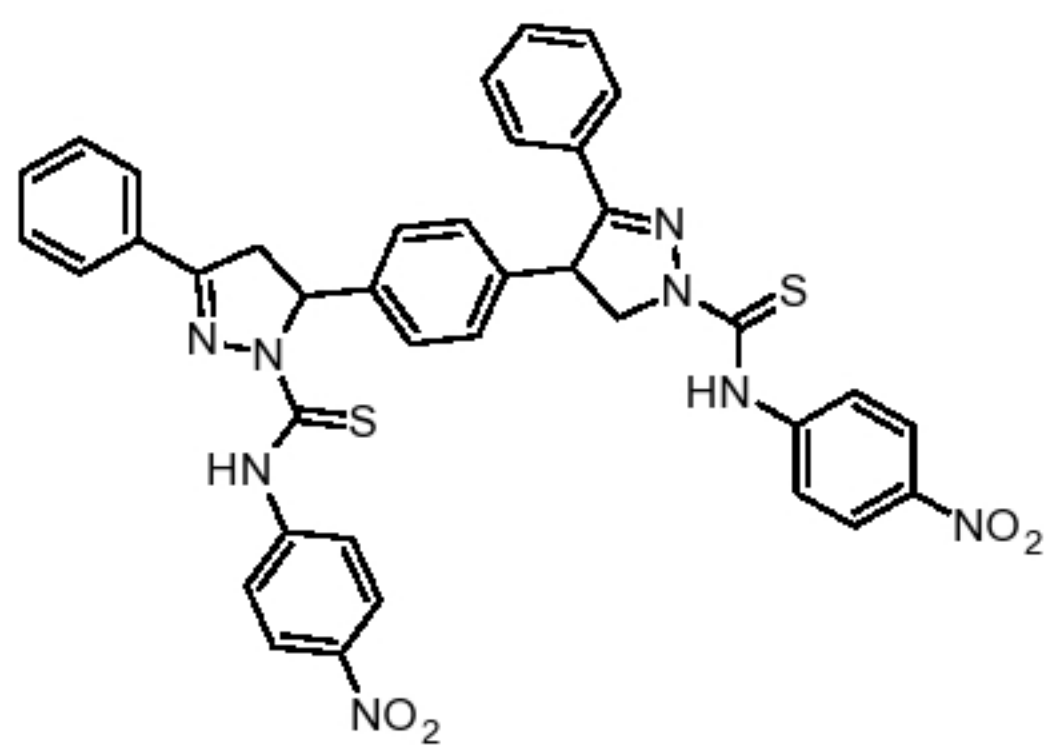

32

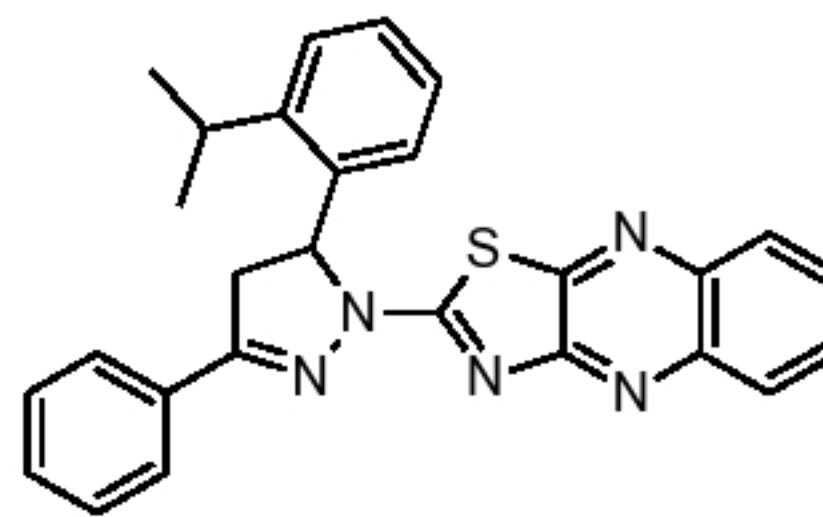

33

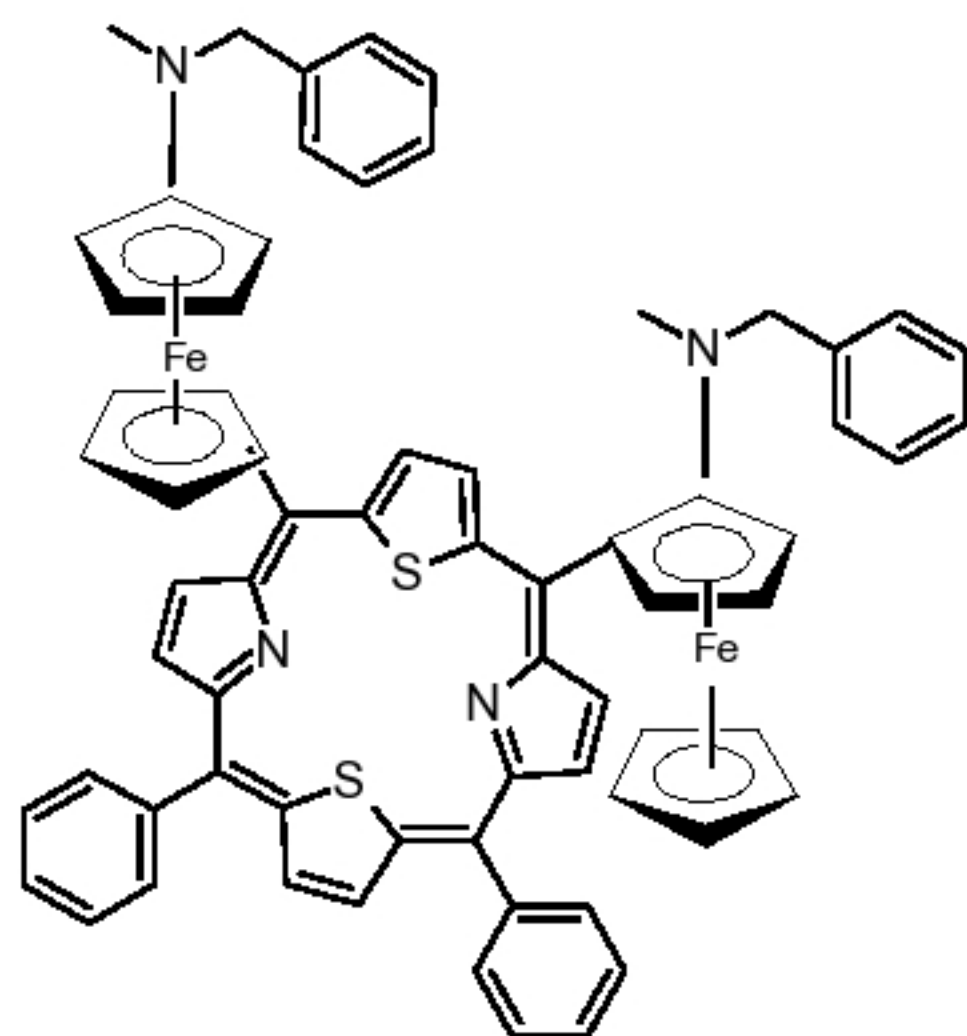

34

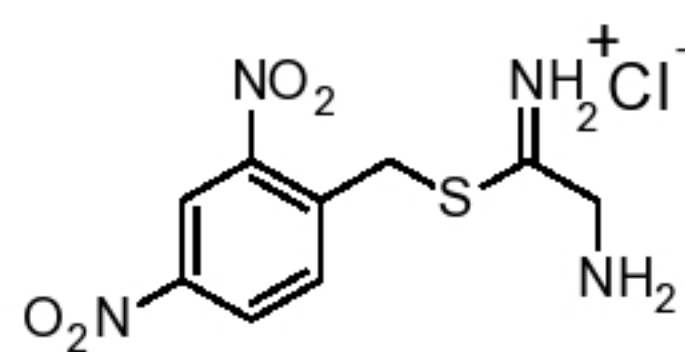

35

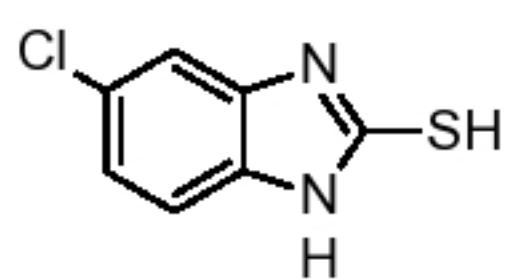

36

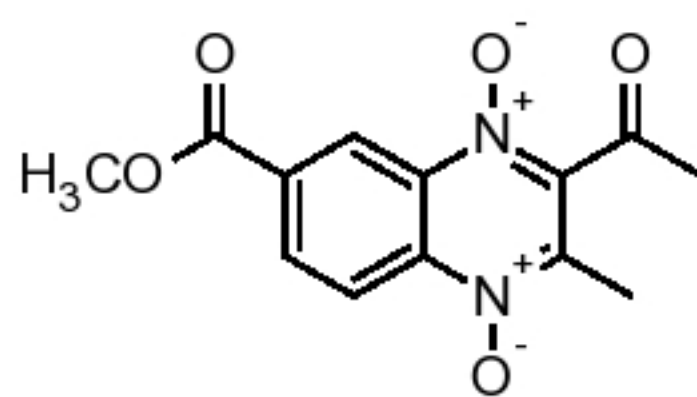

37

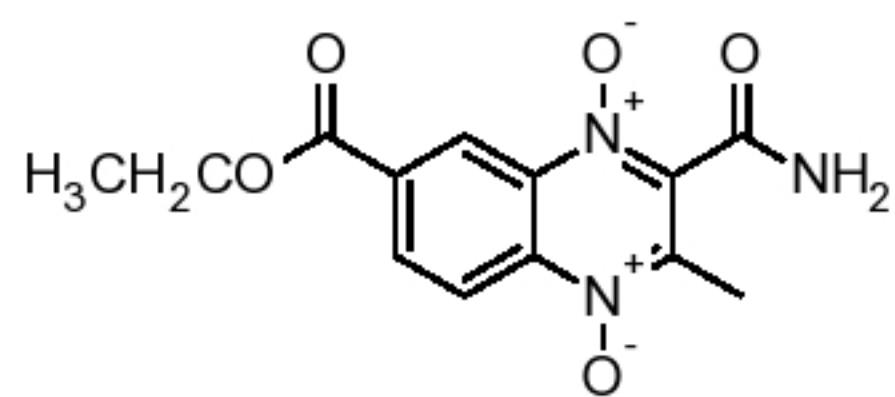

38

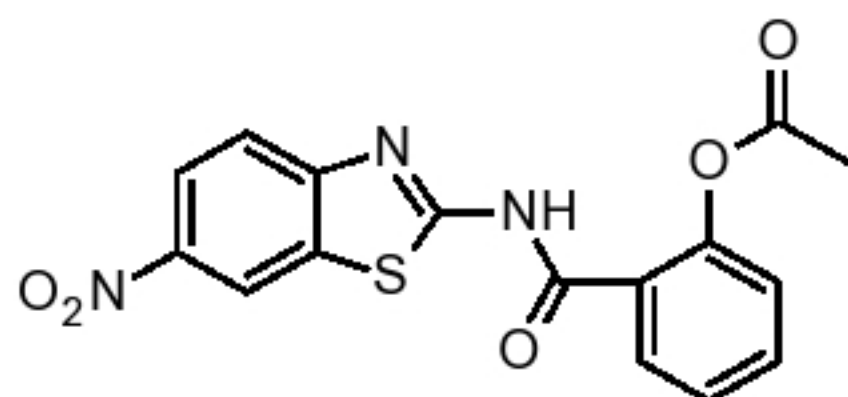

39

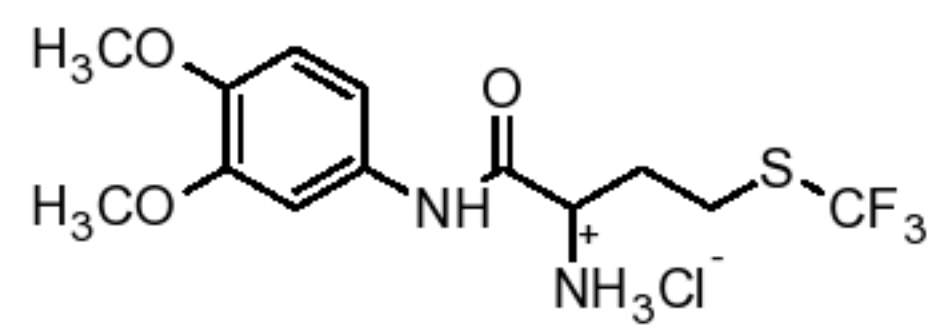

40

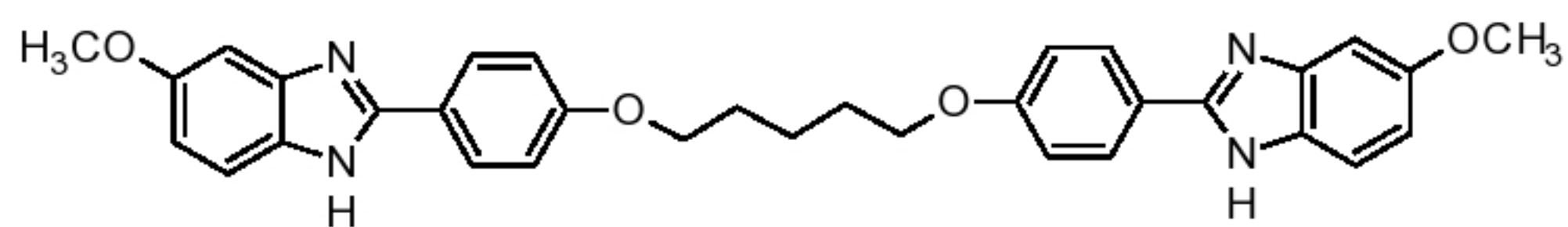

41

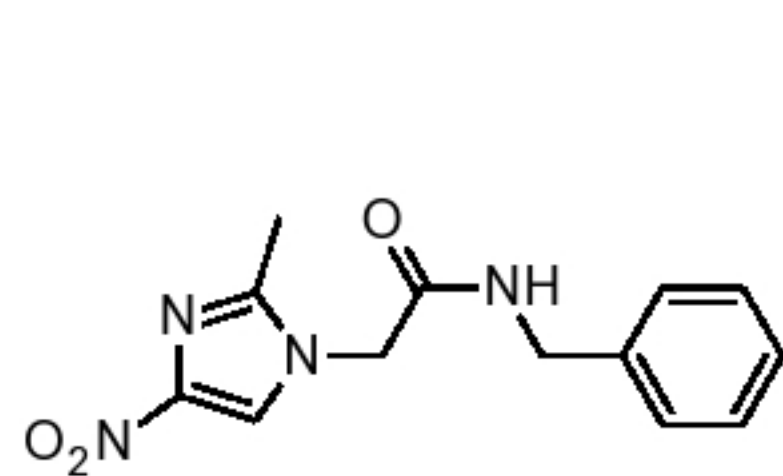

42

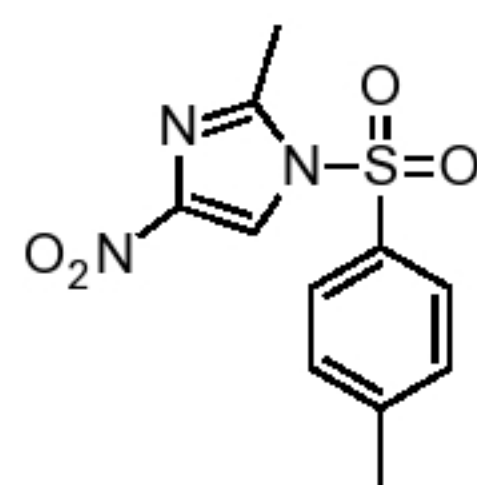

43

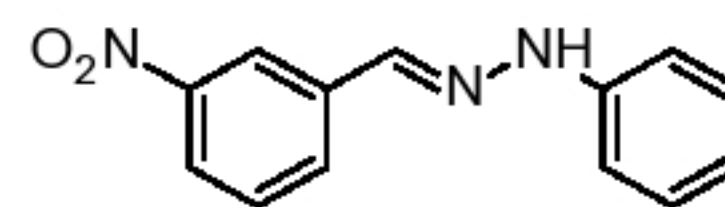

44

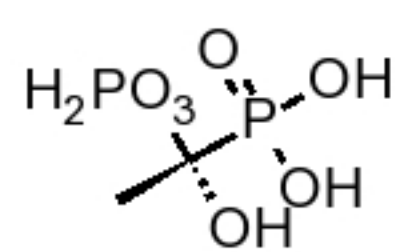

45

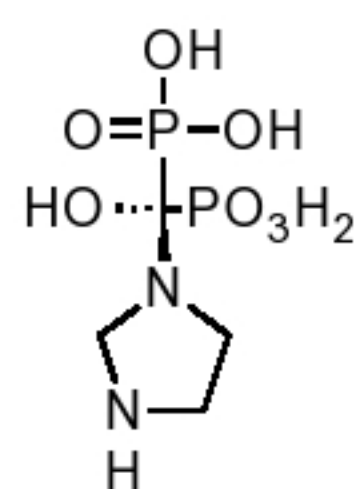

46

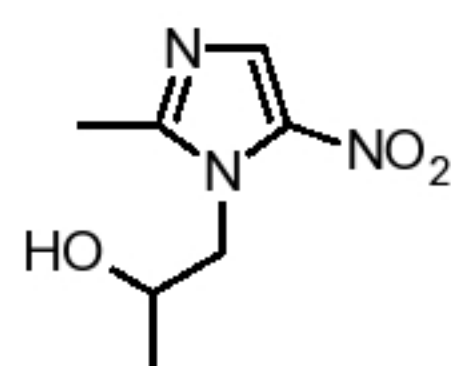

47

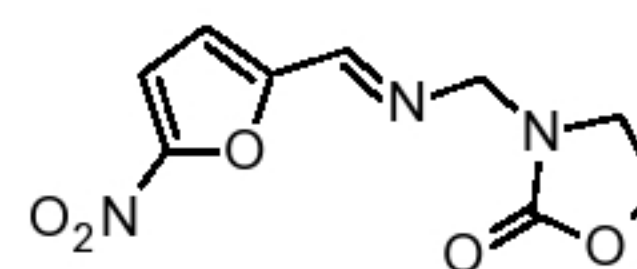

48

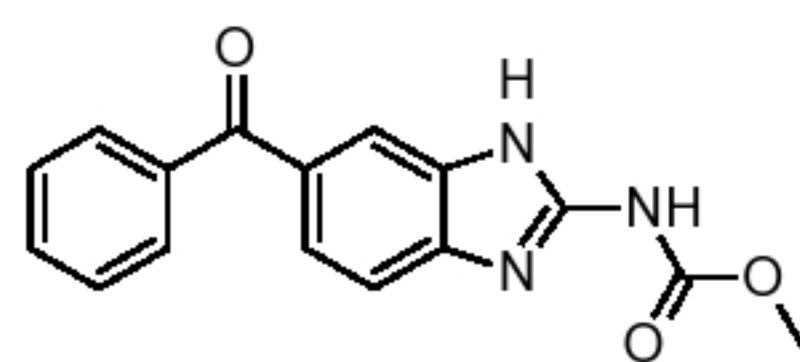

49

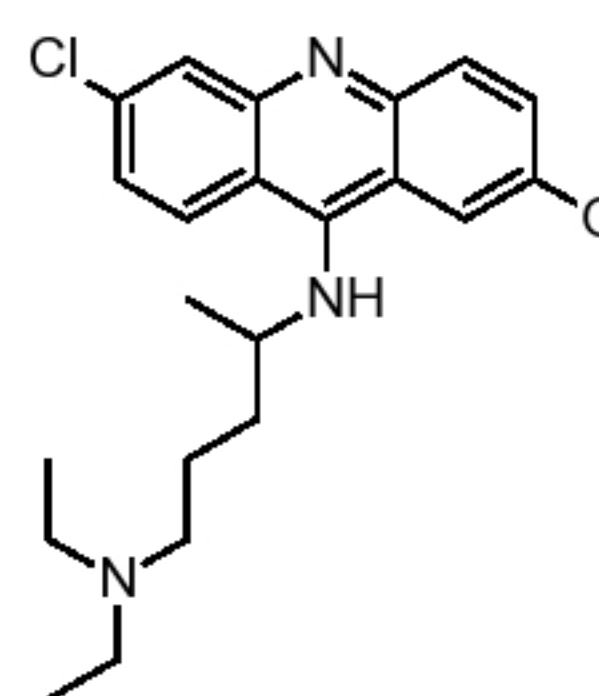

50

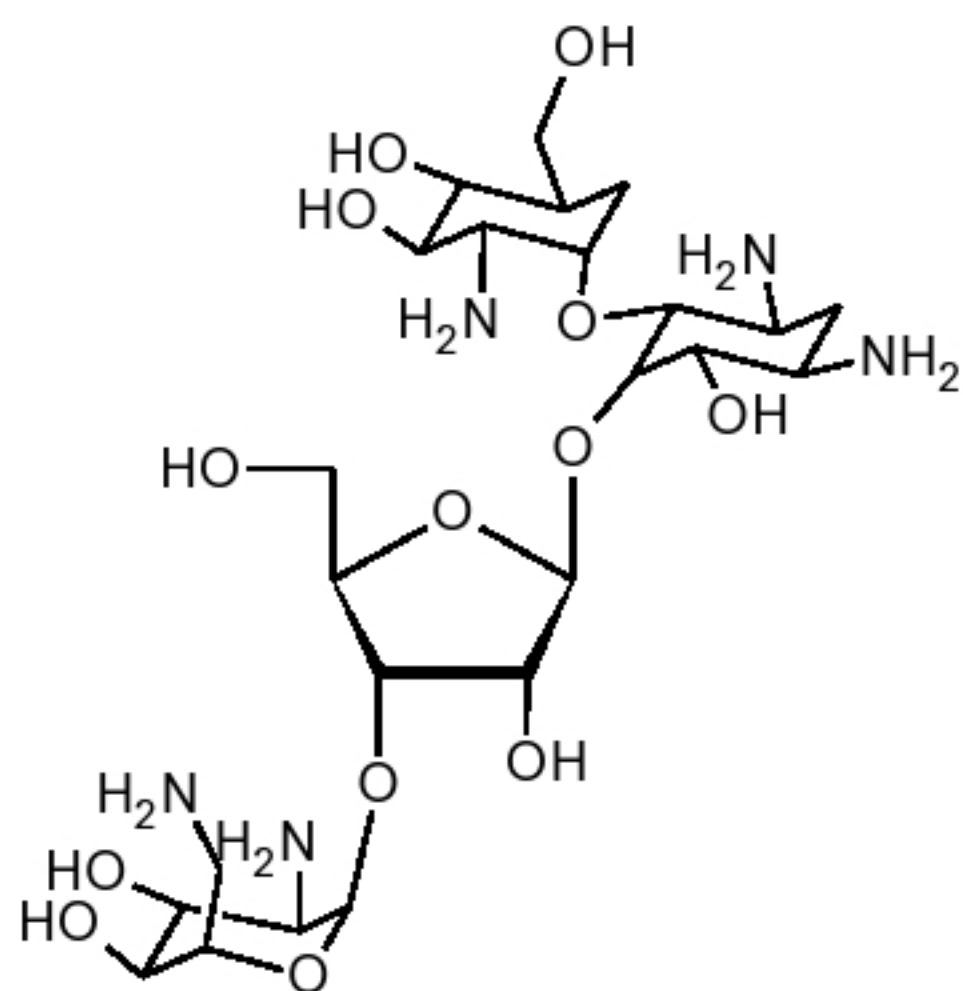

51

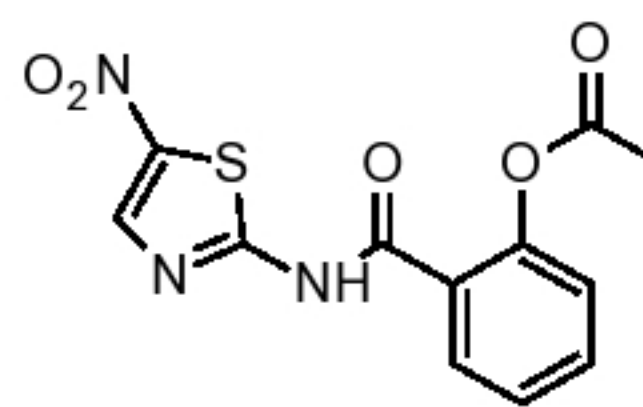

52

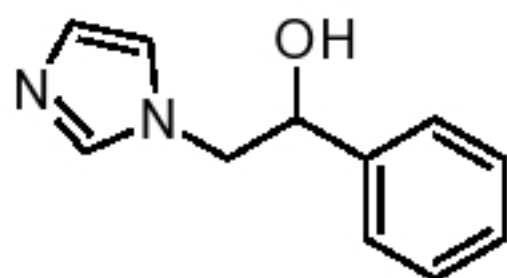

53

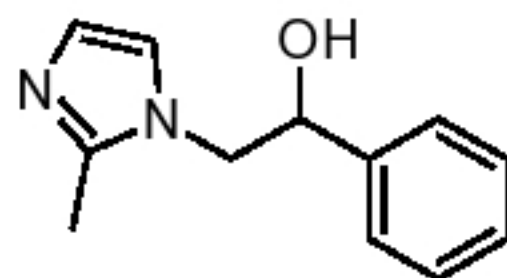

54

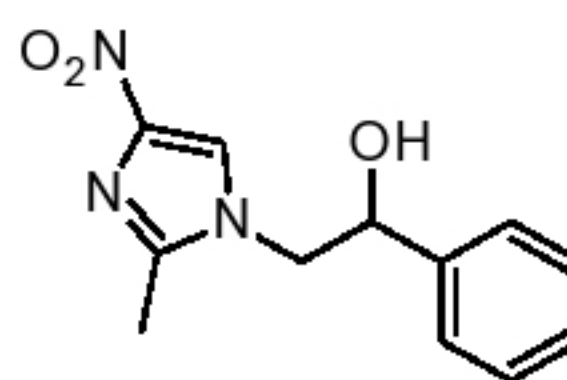

55

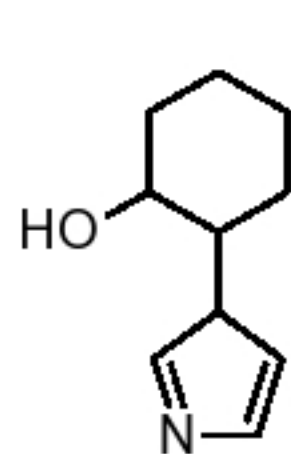

56

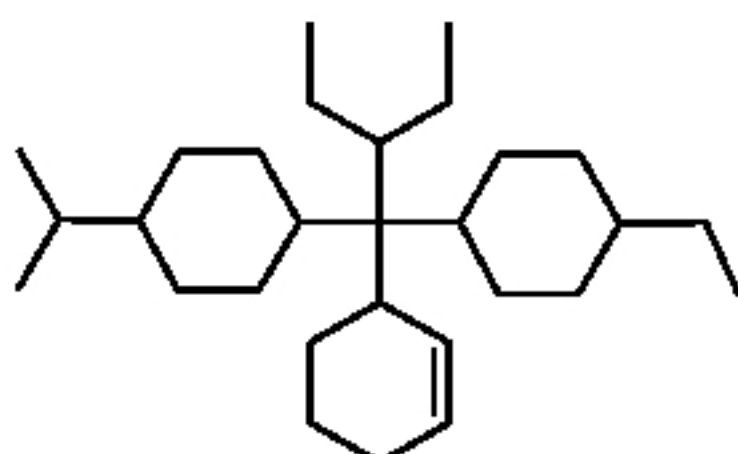

57

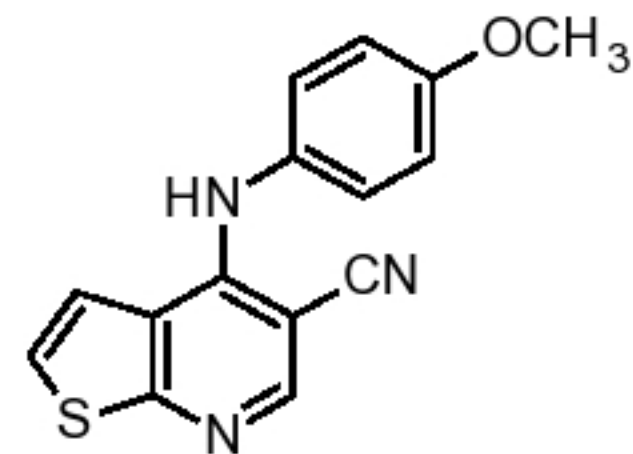

58

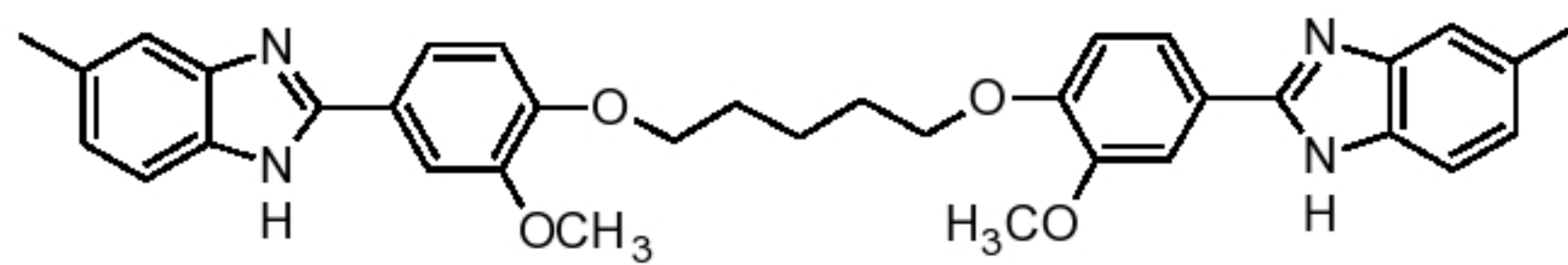

59

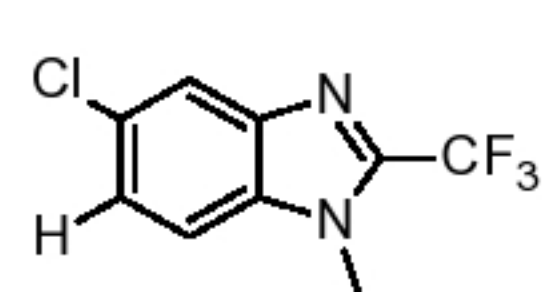

60

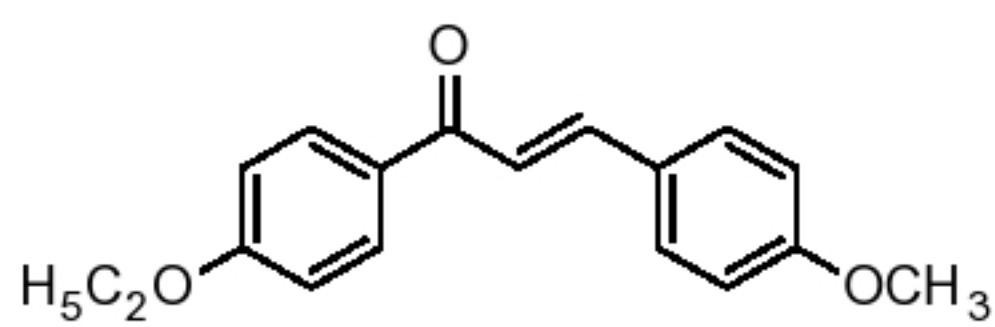

61

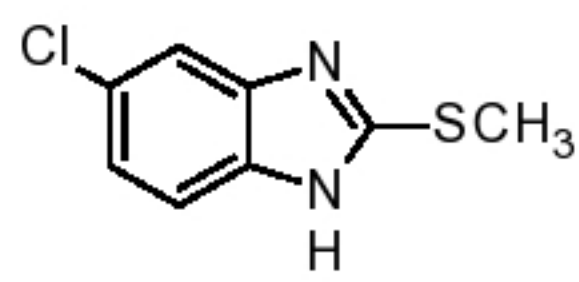

62

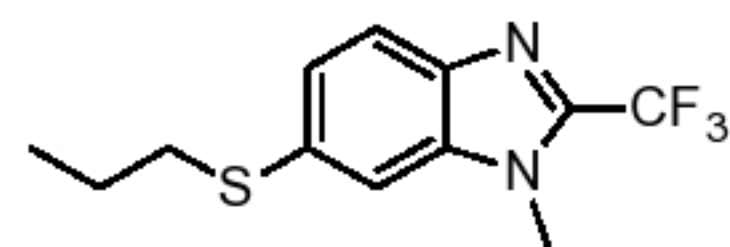

63

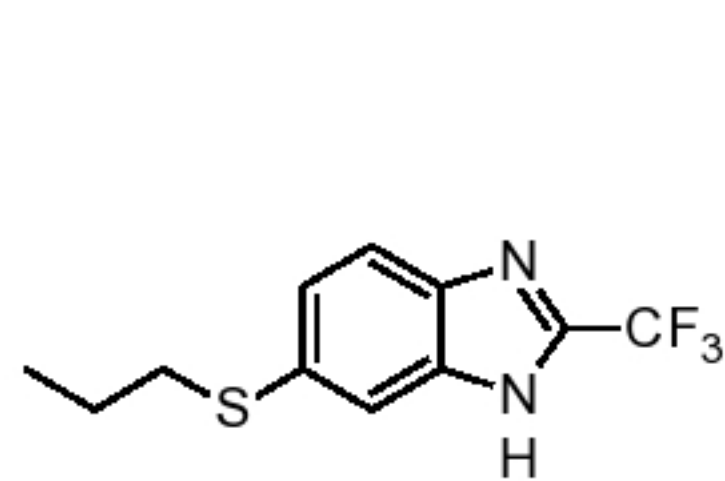

64

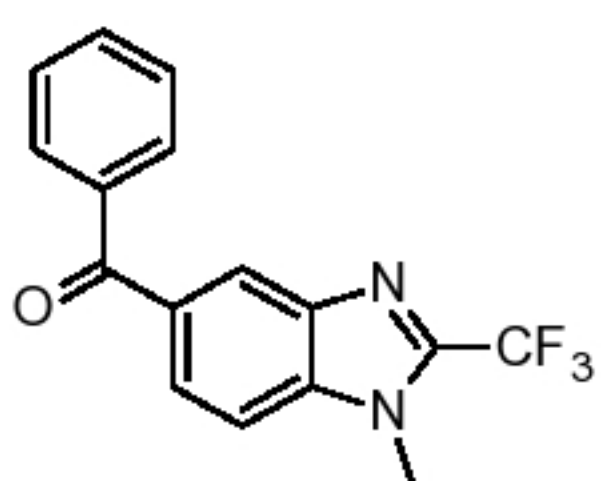

65

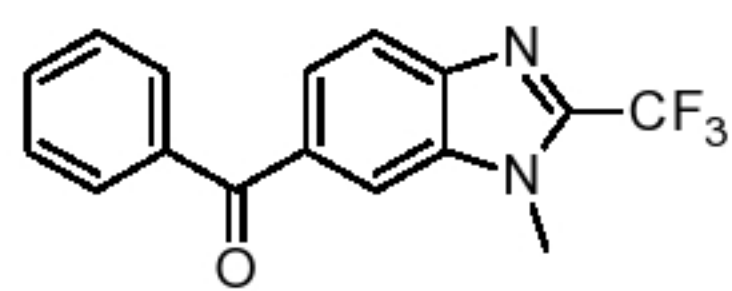

66

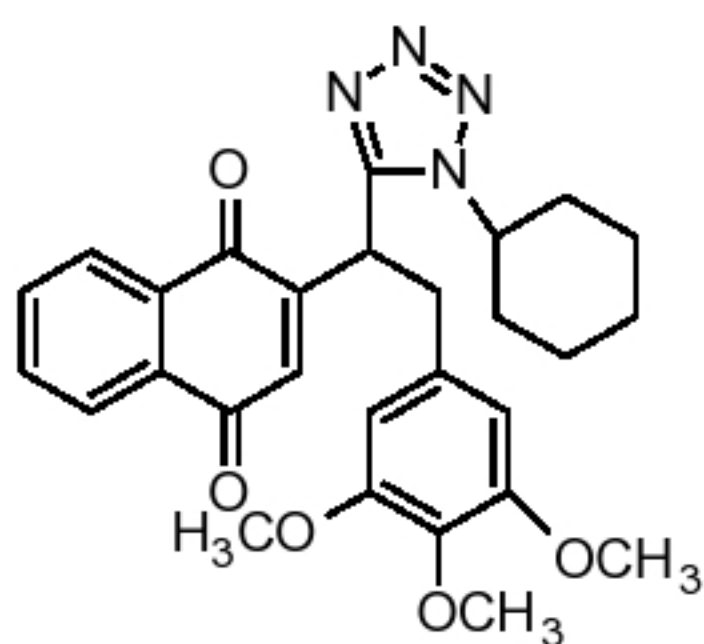

67

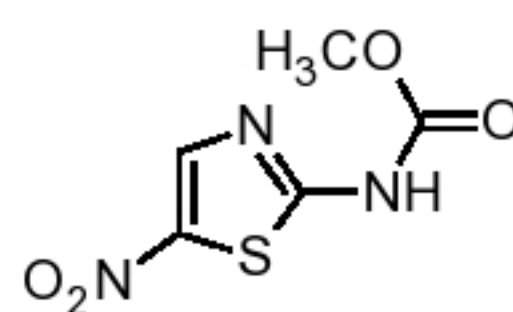

68

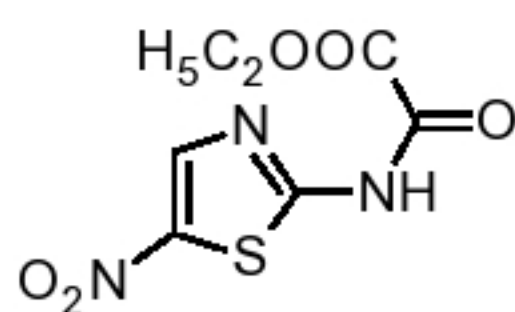

69

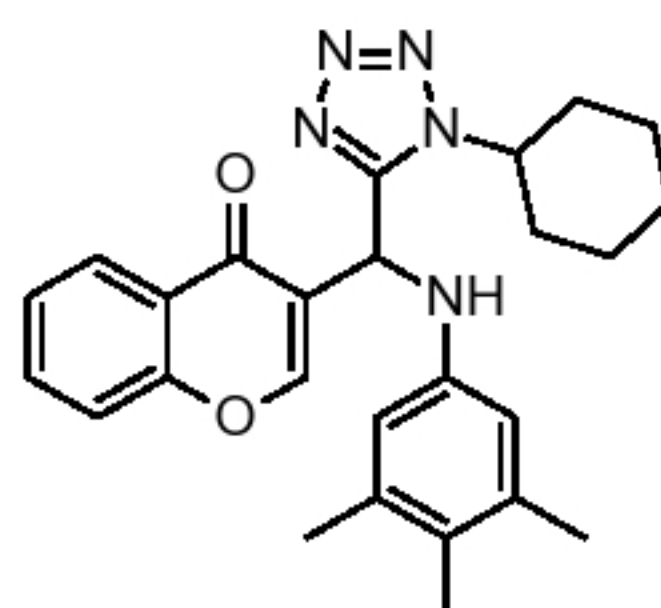

70

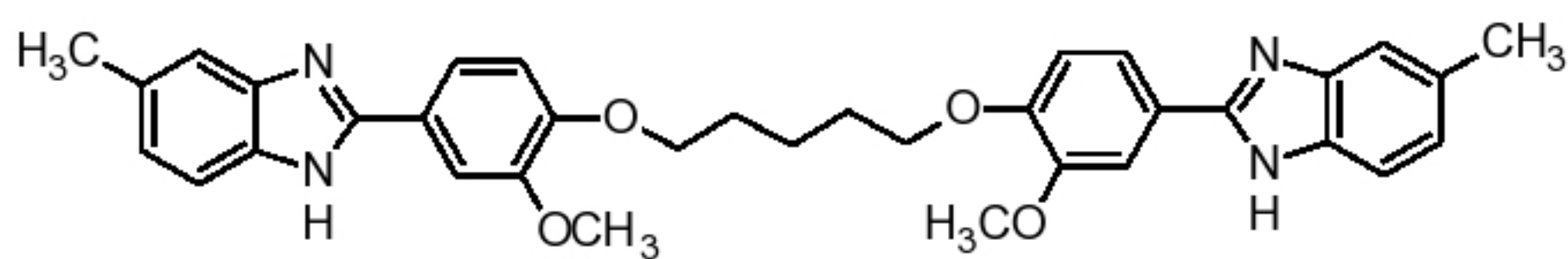

71

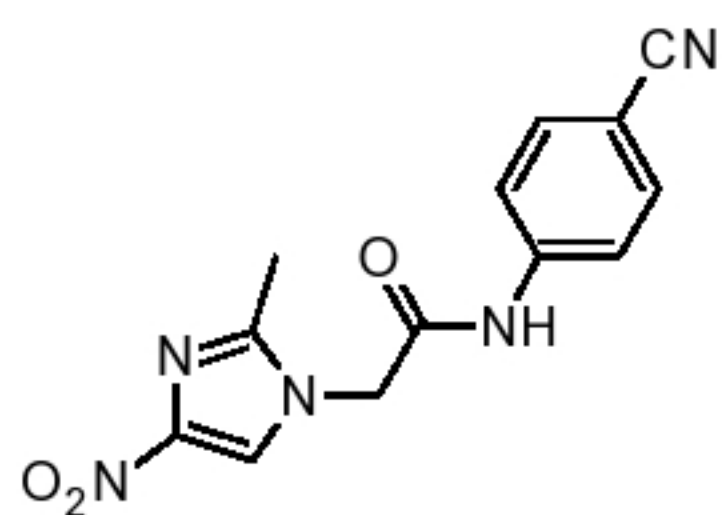

72

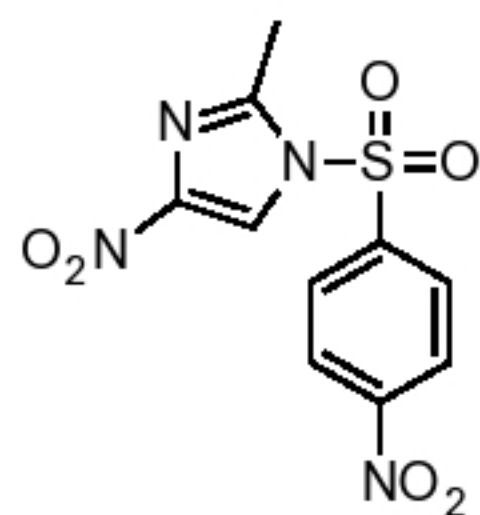

73

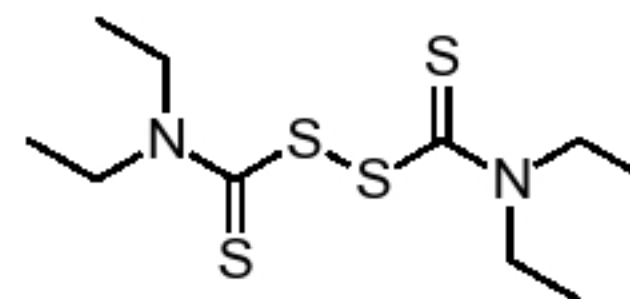

74

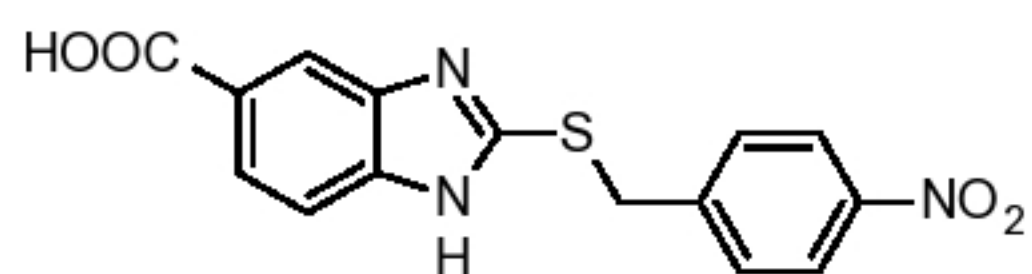

75

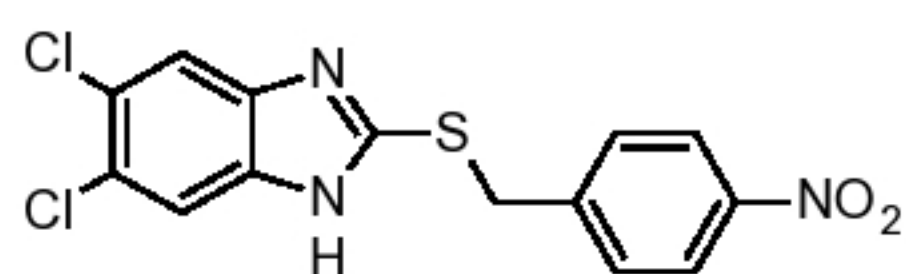

76

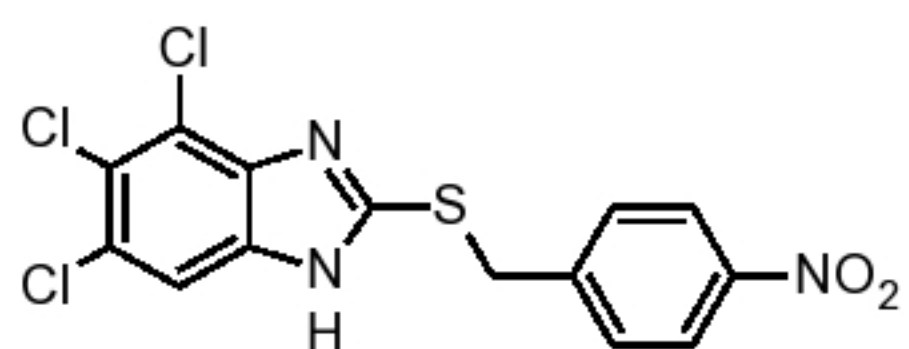

77

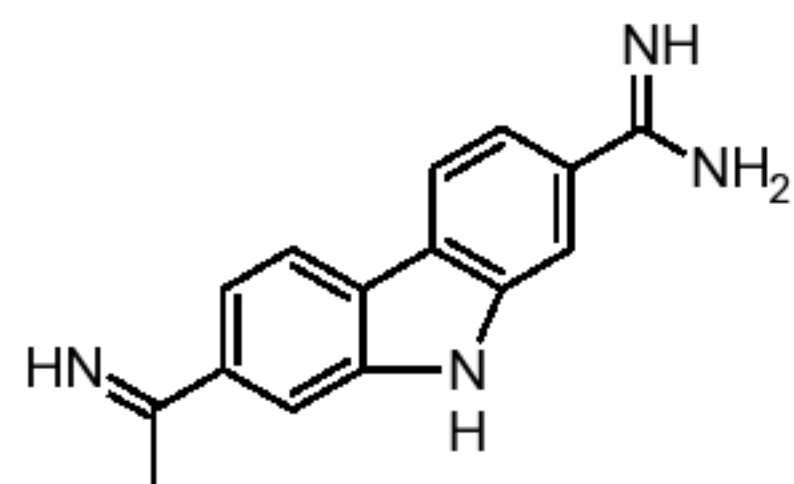

78

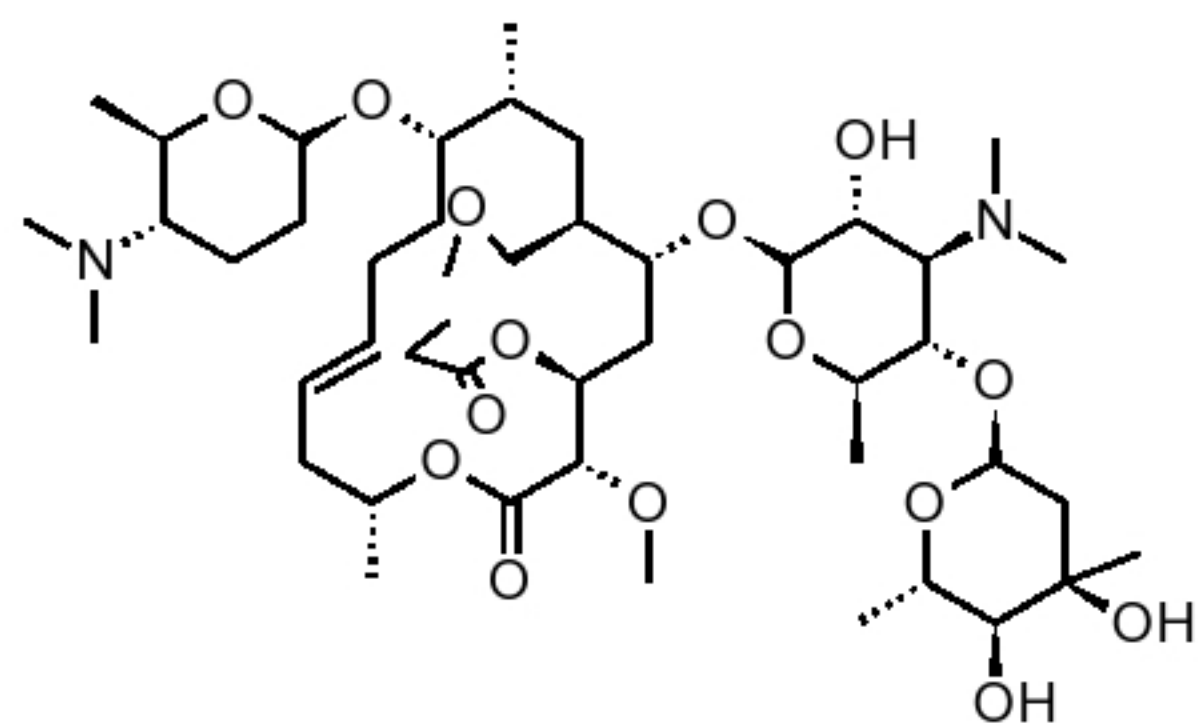

79

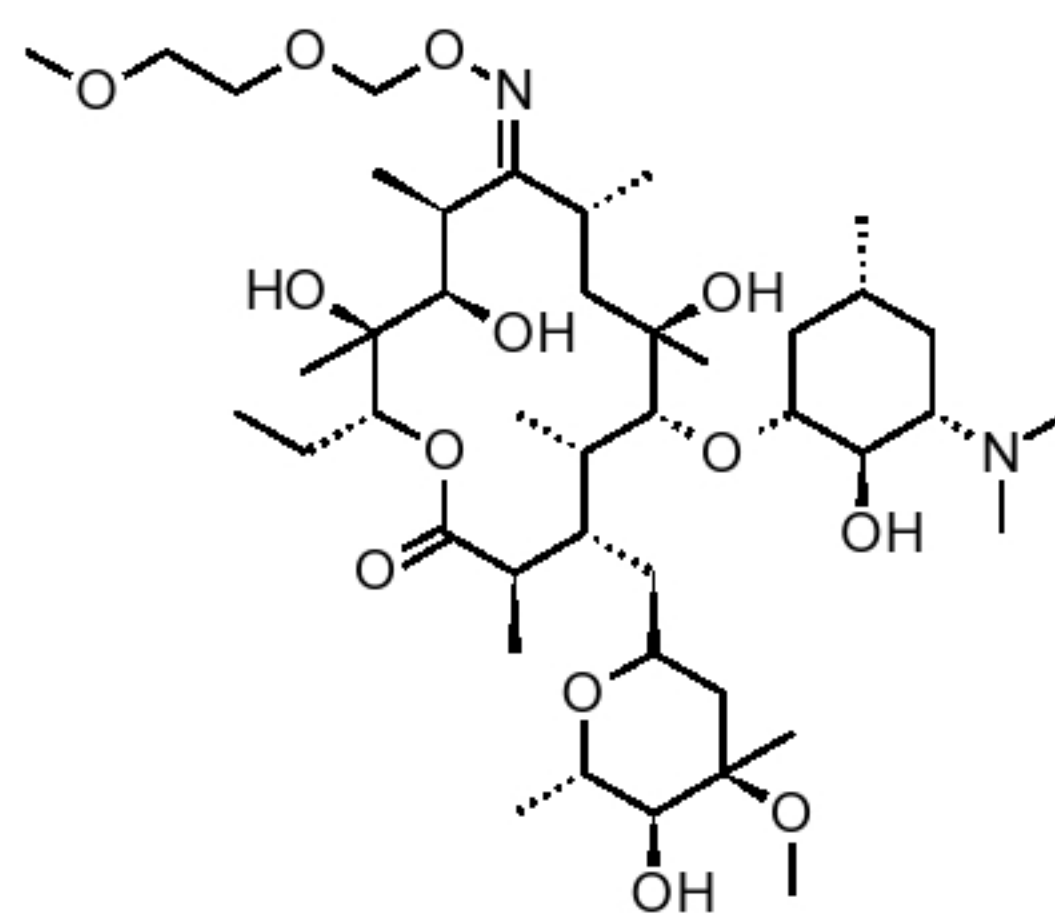

80

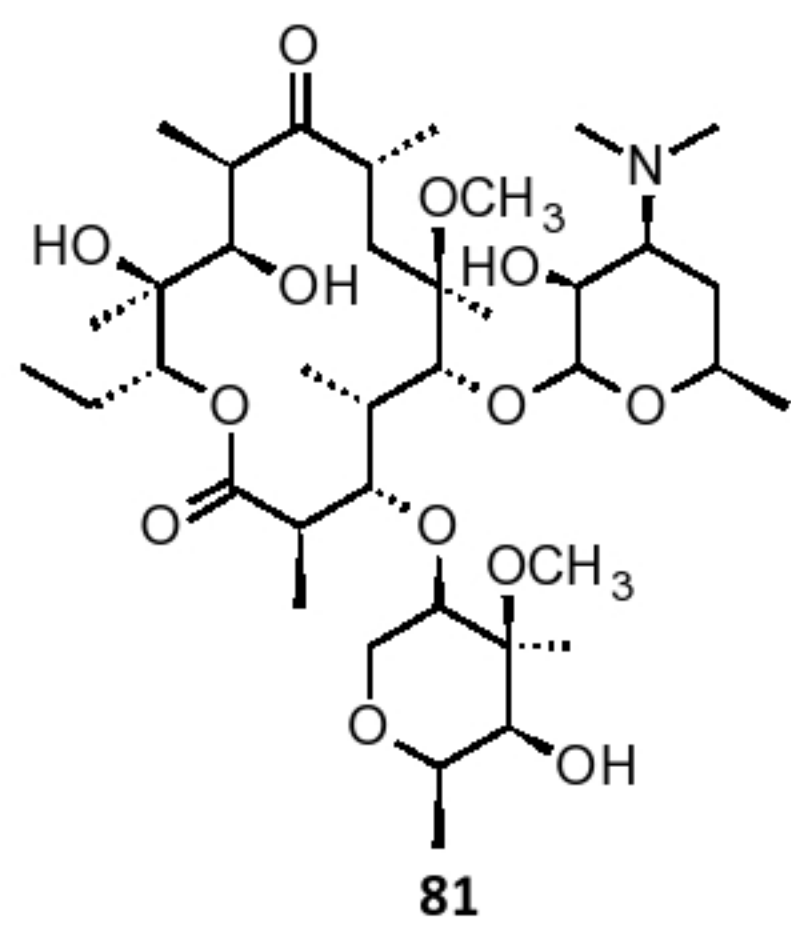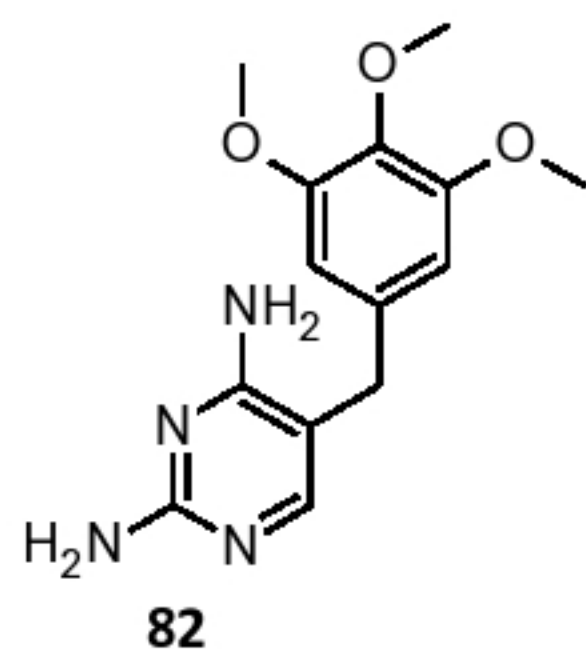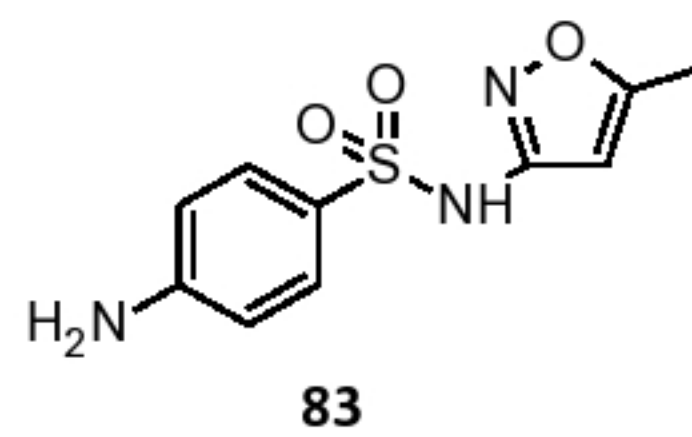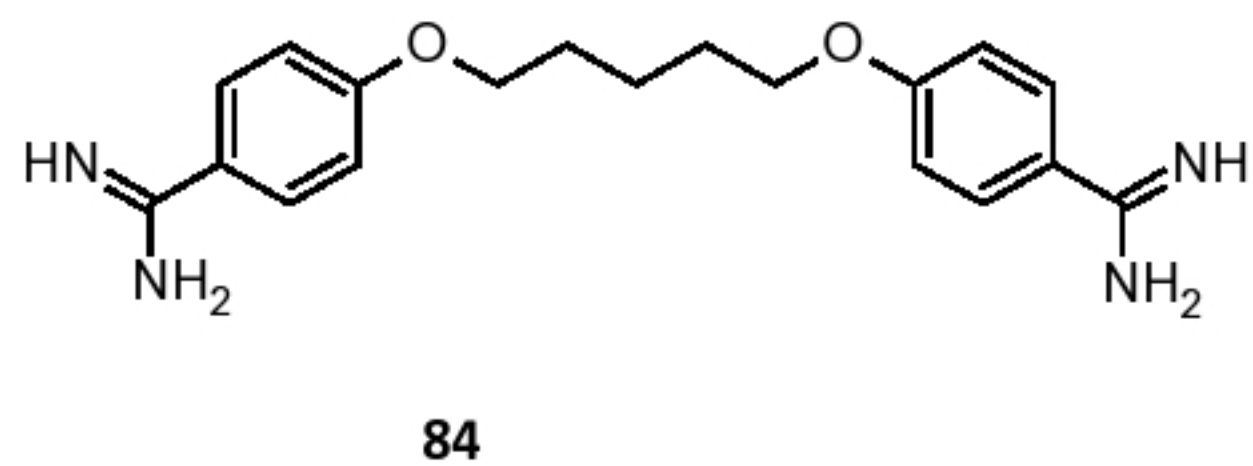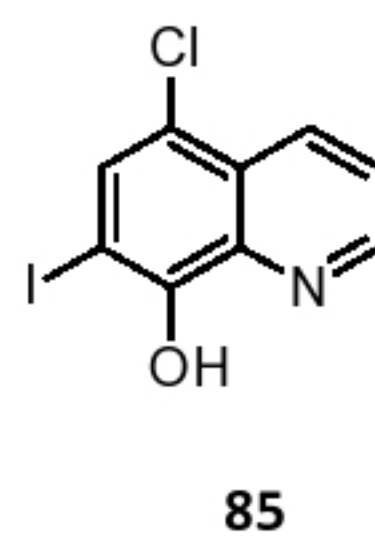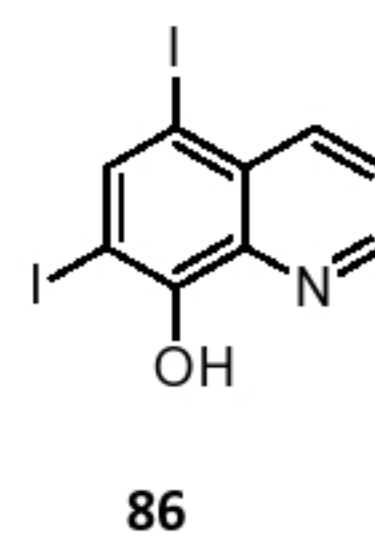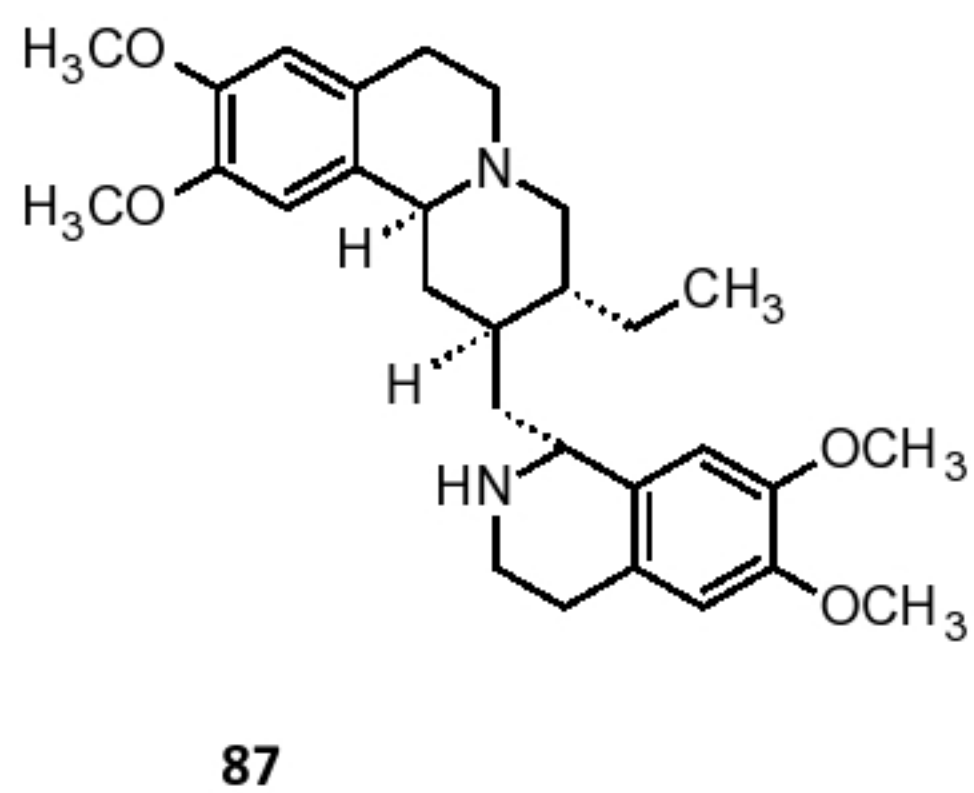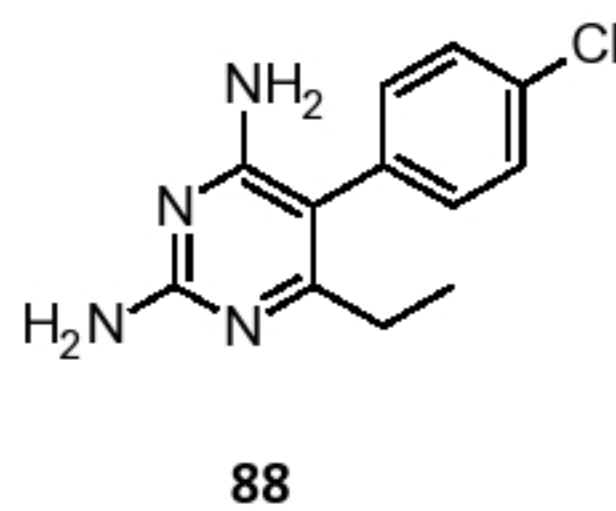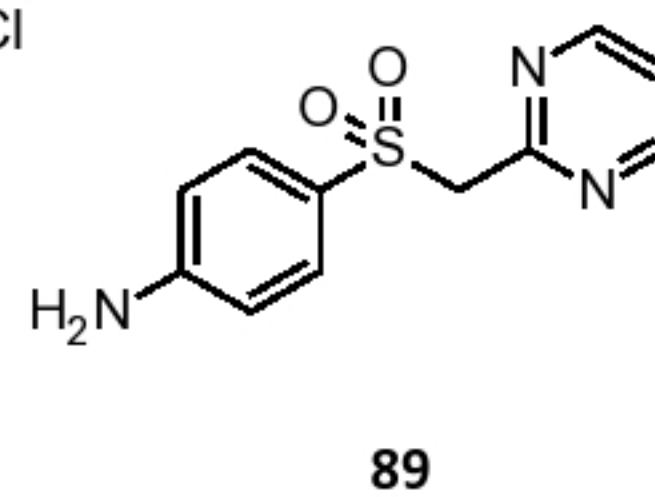

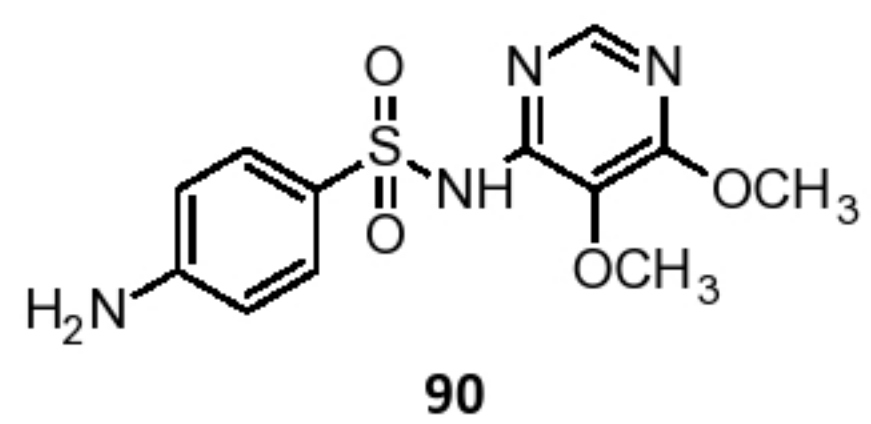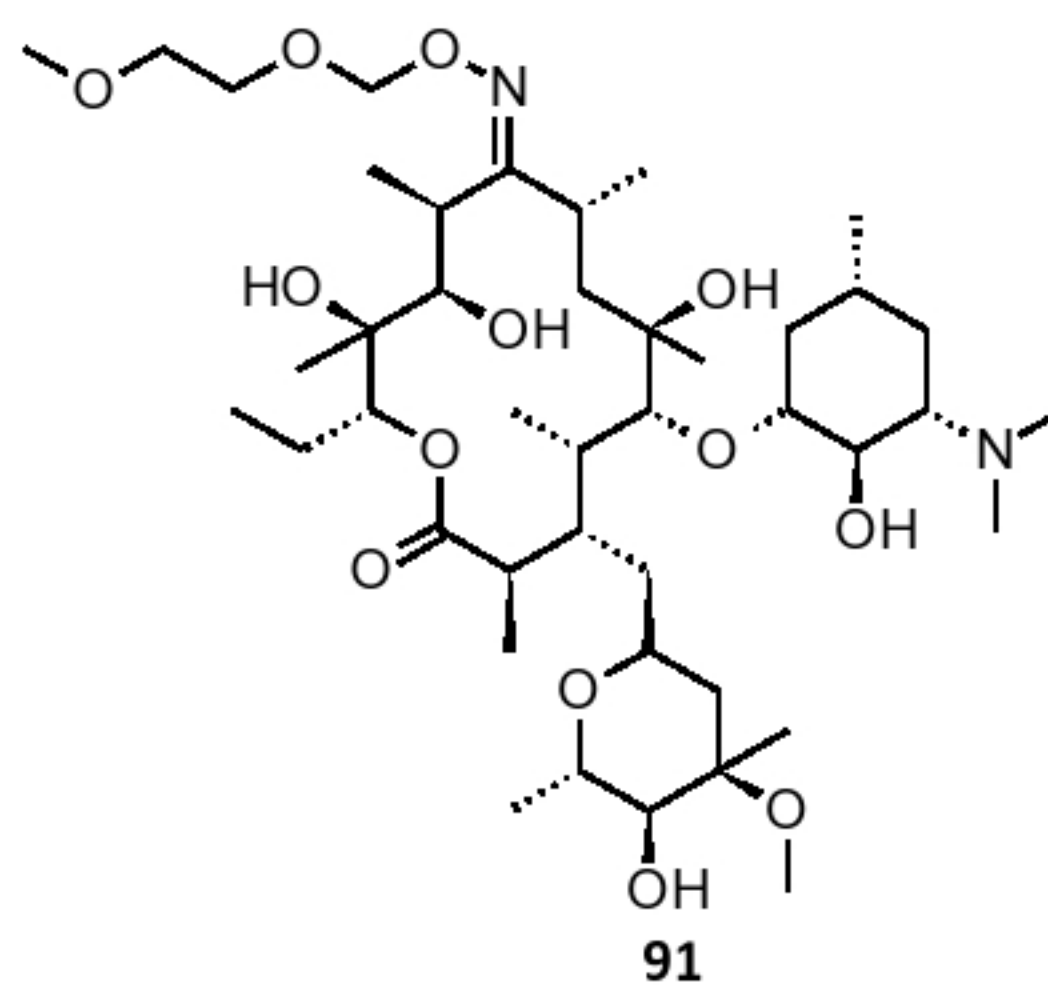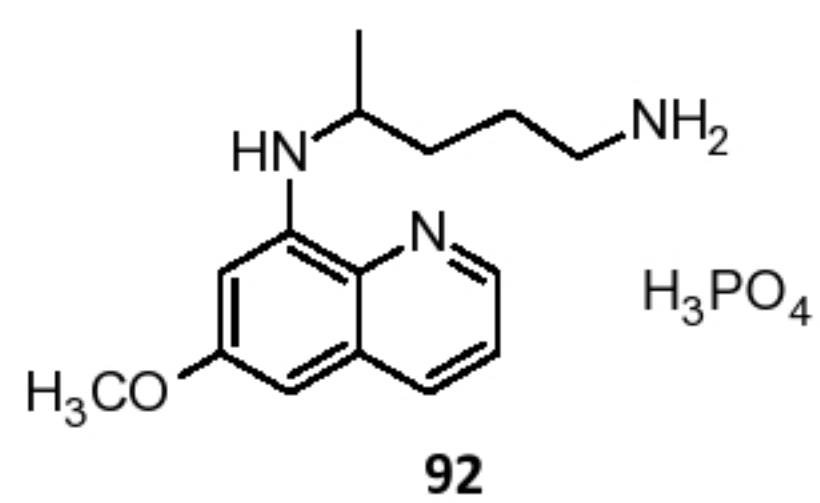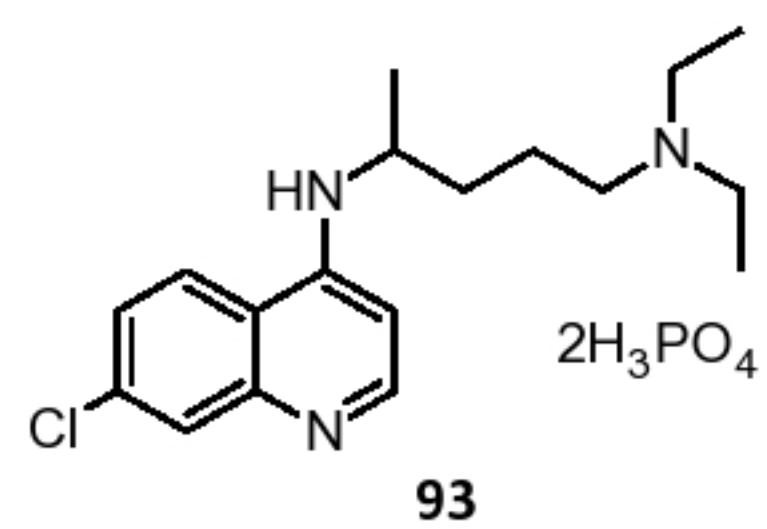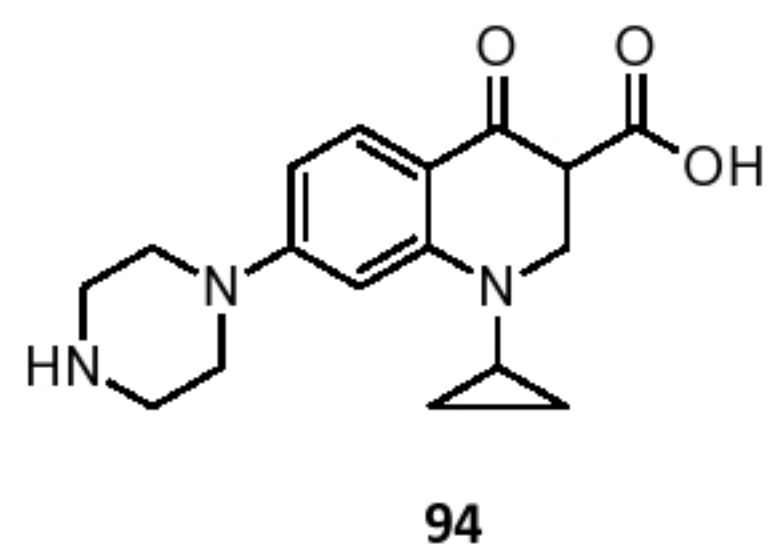

Supplement: Supplementary file 1 [file DataSheet1.PDF]
